# Temperature Control of Fimbriation Circuit Switch in Uropathogenic *Escherichia coli*: Quantitative Analysis via Automated Model Abstraction

**DOI:** 10.1371/journal.pcbi.1000723

**Published:** 2010-03-26

**Authors:** Hiroyuki Kuwahara, Chris J. Myers, Michael S. Samoilov

**Affiliations:** 1 Ray and Stephanie Lane Center for Computational Biology, Carnegie Mellon University, Pittsburgh, Pennsylvania, United States of America; 2 Department of Electrical and Computer Engineering, University of Utah, Salt Lake City, Utah, United States of America; 3 QB3: California Institute for Quantitative Biosciences, University of California, Berkeley, Berkeley, California, United States of America; University of California, Santa Barbara, United States of America

## Abstract

Uropathogenic *Escherichia coli* (UPEC) represent the predominant cause of urinary tract infections (UTIs). A key UPEC molecular virulence mechanism is type 1 fimbriae, whose expression is controlled by the orientation of an invertible chromosomal DNA element—the *fim* switch. Temperature has been shown to act as a major regulator of *fim* switching behavior and is overall an important indicator as well as functional feature of many urologic diseases, including UPEC host-pathogen interaction dynamics. Given this panoptic physiological role of temperature during UTI progression and notable empirical challenges to its direct *in vivo* studies, *in silico* modeling of corresponding biochemical and biophysical mechanisms essential to UPEC pathogenicity may significantly aid our understanding of the underlying disease processes. However, rigorous computational analysis of biological systems, such as *fim* switch temperature control circuit, has hereto presented a notoriously demanding problem due to both the substantial complexity of the gene regulatory networks involved as well as their often characteristically discrete and stochastic dynamics. To address these issues, we have developed an approach that enables automated multiscale abstraction of biological system descriptions based on reaction kinetics. Implemented as a computational tool, this method has allowed us to efficiently analyze the modular organization and behavior of the *E. coli* fimbriation switch circuit at different temperature settings, thus facilitating new insights into this mode of UPEC molecular virulence regulation. In particular, our results suggest that, with respect to its role in shutting down fimbriae expression, the primary function of FimB recombinase may be to effect a controlled down-regulation (rather than increase) of the ON-to-OFF *fim* switching rate via temperature-dependent suppression of competing dynamics mediated by recombinase FimE. Our computational analysis further implies that this down-regulation mechanism could be particularly significant inside the host environment, thus potentially contributing further understanding toward the development of novel therapeutic approaches to UPEC-caused UTIs.

## Author Summary

Urinary tract infections (UTIs) represent a major growing threat to global public health. With over 15 million cases a year in the United States alone, UTIs are characterized by very high recurrence/reinfection rates, particularly among women and minority groups [1]. The predominant cause of UTIs is uropathogenic *Escherichia coli* (UPEC) bacteria, whose wide-spread and increasing antibiotic-resistance has made the development of alternative anti-UPEC treatments progressively more important and urgent. UPEC's foremost virulence factor is hair-like surface structures called *type 1 fimbriae*. Thus, one such potentially promising therapeutic approach may be to manipulate bacteria's own cellular circuitry toward inducing UPEC to turn off their fimbriae expression—rendering individual microbes benign. This task requires detailed understanding of molecular mechanisms involved, which may be significantly aided by *in silico* modeling. However, for UPEC fimbriation control circuit and many other systems, low-level all-inclusive quantitative models inevitably become too computationally demanding to remain practical, while high-level qualitative representations frequently prove inadequate owing to the substantial organizational and behavioral complexity of biological processes involved.

We have developed an automated multiscale model abstraction methodology that helps address these problems by systematically generating intermediate-level representations that rigorously balance computational efficiency and modeling accuracy. Here, we use such an approach to examine how different temperature settings quantitatively affect UPEC transitions between fimbriate and afimbriate phases, to gain new understanding of the underlying modular circuit switch control logic, and to suggest further insights into ways this knowledge could potentially be used in therapeutic applications.

## Introduction

Type 1 fimbriae (pili) represent the foremost virulence factor in lower urinary tract infections (UTIs) by uropathogenic *Escherichia coli* (UPEC)—the main causative agent that accounts for 80–90 percent of all community-acquired UTIs in the United States [1]–[4]. These adhesive surface organelles have been identified as both the UPEC virulence factor most frequently found in clinical isolates as well as the one that experiences the highest absolute and among the greatest relative increases of component gene expression *in vivo* during UTIs [5],[6]. Type 1 fimbriae also have been shown to fulfill molecular Koch's postulates [2],[7] and have been further reported as the only major uropathogenic virulence factor that is broadly significant for enteric *E. coli* strains as well [8],[9]. The hair-like structures involved vary from a few fractions of a micrometer to more than 3 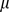
m in length and consist of 7nm-thick right-handed helical rods—largely made up of repeating 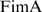 subunits—with 3nm-wide tips containing the 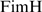 adhesin, which can bind to D-mannose-containing residues found on the surface of epithelial cells and mediate their invasion by UPEC [10]–[13]. Type 1 fimbriae are further thought to aid the UPEC infection process by enhancing the ability of bacteria to form biofilms and to develop intracellular bacterial communities (IBCs) with biofilm-like properties [13]–[18]. The latter allow *E. coli* to establish quiescent pathogen reservoirs shielded from native host defenses and antibiotic treatments as well as serve to seed subsequent UTIs in a type 1 fimbriae-dependent manner [2], [13], [19]–[21]. This may both contribute to the widespread emergence of multi-drug-resistant UPEC strains (up to 20–50 percent of isolates) as well as help account for the notably high rates of UTI incidence (lifetime risk of over 50 percent for women and nearly 14 percent for men) and recurrence (40 percent in women and 26 percent in men per annum) – along with leading to a number of other significant public health implications (e.g., over 10 million estimated annual physician office visits in the United States alone) [1],[22]. However, while they provide a means for infection, type 1-fimbriated UPEC populations also have lower fitness due to phase-specific mechanisms that directly decrease growth rates through additional costs of fimbriae synthesis and contact-dependent inhibition as well as reduce motility, which allows competitors to more efficiently occupy advantageous colonization sites and take up resources [6], [23]–[25]. Furthermore, type 1 fimbriae-mediated attachment can lead to preferential exfoliation of infected cells as part of the host immune response, which can result in rapid clearance of the infection [13], [20], [26]–[28]. Among other things, this apparent dichotomy between the essential role played by the piliated phase in the establishment of the infection and the noted fitness disadvantages conferred upon individual bacteria by type 1 fimbriae implies that their expression needs to be highly optimized and tightly controlled.

**Figure 1:**
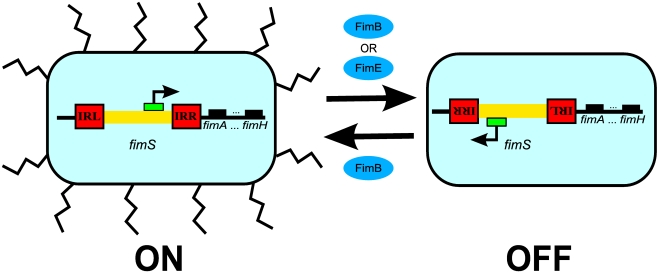
Phase variation of type 1 fimbriae expression in *E. coli*. Type 1 fimbriae phase variation is controlled by the invertible DNA element, *fimS*, which contains the promoter for the genes encoding structural fimbriae subunits (including *fimA* and *fimH*) and is flanked by two inverted repeat sequences: IRL and IRR. (In this diagram, IRL is the inverted version of IRR.) When the switch is in the ON position, transcription of structural *fim* genes can be initiated because the promoter is in the appropriate orientation. However, when the switch is inverted into the OFF position, the promoter points in the opposite direction and so no longer supports the expression of fimbriae components—leading to their rapid degradation. The ON-to-OFF inversion of the switch is mediated by recombinases FimE and FimB, while the OFF-to-ON events are mediated by FimB.

As illustrated in Figure 1, the expression of type 1 fimbriae in *E. coli* is *randomly* phase variable, whereby individual cells stochastically switch between *fimbriate* (ON) and *afimbriate* (OFF) states with rates regulated by various internal as well as environmental conditions [29]–[33]. With the ongoing advancements in high-resolution single-cell and single-molecule scale experimental methods, such bimodal and bistable mechanisms for generating phenotypic heterogeneity in clonal cell populations have been increasingly often identified and investigated across a broad range of prokaryotic and eukaryotic systems—where they have been shown to influence a diverse spectrum of processes—including organism development, behavior, disease, survival, and memory [34]–[44]. In the case of *E. coli* type 1 fimbriae, this phase variation is controlled by the *fim* circuit switch that functions based on the inversion of a 314bp chromosomal region, *fimS*, bounded by two 9bp inverted repeats left and right (IRL and IRR) [29],[34],[45],[46]. The *fimS* element contains the promoter for *fimA* and other genes encoding structural subunits of type 1 fimbriae. As a result, an individual *E. coli* cell expresses type 1 fimbriae when the *fim* switch is in the ON position and rapidly becomes afimbriate when the switch flips into the OFF position [34],[47]. This inversion of *fimS* requires either 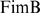 or 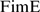 site-specific recombinases binding at IRL and IRR [29],[47],[48]. However, whereas 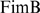 mediates recombination with little orientational bias, 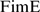 mediates recombination predominantly in the ON-to-OFF direction [30],[49]. Empirical evidence has further revealed that the inversion of the *fim* switch is strongly controlled by temperature in a complex manner [30],[31]. In particular, observations at 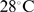, 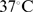, and 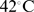 have indicated that wild-type ON-to-OFF switching frequency—dominated by 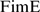
—decreases in an exponential-like fashion as temperature increases, while 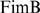-mediated switching frequency is higher at 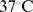 than either at 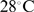 or 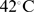 in both defined-rich and minimal media. Experimental results also show that the wild-type ON-to-OFF switching rate is much faster than 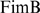-mediated switching rate alone, allowing *E. coli* to rapidly undergo afimbriation under appropriate conditions [30],[50].

This work investigates the logic and behavior of the gene regulatory circuit, which controls the ON/OFF switching of type 1 fimbriae expression, by starting with the reaction-level description of its underlying biochemical and biophysical molecular interaction mechanisms. We are particularly interested in the role of environmental cues in this process and, specifically, of temperature as it is known to control many gene regulatory circuits in bacteria—often those responsible for virulence functions [51]. Temperature variations are also frequently characteristic of host-pathogen interaction dynamics—such as during cytokine response (e.g., through IL-6 as well as IL-8 and IL-1) and the ensuing inflammation that is indicative of the onset and progression of UPEC UTIs—as well as often generally representative of urinary tract pathology [52],[53]. In this context, reaction-level modeling provides a framework for highly accurate description of the underlying biomolecular circuit behavior through application of the corresponding fundamental chemical and physical principles. However, the innate complexity of biological networks involved as well as the key role played by nonlinear, discrete, and stochastic kinetics in regulating the dynamics of cellular pathways driven by molecular-scale mechanisms result in profound computational challenges to their accurate quantitative analysis. The problem becomes particularly acute when dealing with biological systems, such as type 1 fimbriation circuit switch dynamics in UPEC, whose behavior is driven by internal or external discrete-stochastic processes to exhibit qualitative deviations from what might otherwise be expected on the bases of “classical” continuous-deterministic biochemical modeling via mass-action kinetics and reaction rate differential equations [39],[54]. The resulting “deviant” dynamics lead such biological systems to behave in a distinctive but often quite unintuitive manner, which necessitates the use of differential-difference modeling based on the chemical master equation framework (see [54]–[59] and Methods for details). However, while the latter approach is able to accurately account for both the stochastic occurrence as well as the discrete nature of individual molecular interactions that underlie the design, function, and control of most biological circuits—it also tends to produce dramatic increases in the associated analytical and computational demands [60]–[62].

Although these computational limitations may often render any direct implementations of the all-inclusive low-level quantitative models impractical, the use of entirely high-level qualitative representations frequently becomes inadequate as well, owing to the substantial multiscale dynamical and functional complexity that biological systems can manifest. In such cases, *in silico* analysis can greatly benefit from applications of appropriate intermediate-level system *model abstractions*—whereby multiple individual biological interactions are aggregated into significantly few(er), but quantitatively analogous functional processes. An optimized model abstraction scheme then looks to accurately capture the target characteristics of biological system behavior, while trading off some tightly controlled degree of precision for significant computational gains. Additionally, the resulting abstracted model of the system may also be useful in helping to uncover any general high-level logical patterns embedded within the biological networks involved, which can otherwise be obscured by the low-level molecular interaction mechanics.

Our method initiates the abstraction procedure with a detailed reaction-level representation of biological processes in question. This enables it to utilize basic biochemical and biophysical principles to rigorously implement many of the existing as well as potentially allow for the development and incorporation of novel abstraction techniques, Table 1, in order to insure the desired degree of modeling accuracy versus computational efficiency for the abstracted representation at the system scale of interest [63],[64].

**Table 1:** Abstraction methods used by reb2sac in the analysis of the *fim* circuit switch model.

Abstraction method^a^	Entry^b^
*Quasi-steady-state approximation*	abs[2][3]
*Rapid equilibrium approximation*	abs[2][2]
*Production-passage-time approximation*	abs[2][4]
*Dimerization reaction reduction*	abs[2][5]
*Operator site reduction*	abs[2][6]
*Modifier constant propagation*	abs[1][1]
*Similar reaction combination*	N/A
*Kinetic law simplification*	abs[3][1]
*Irrelevant node elimination*	abs[2][1]
*Stoichiometry amplification*	N/A
*Reaction splitizations*	N/A
*Finite state system transformation*	N/A
*N-ary transformation*	N/A

A detailed discussion of the listed abstraction methods can be found in references [63],[64],[138],[139].

^*a*^Most recent version of reb2sac is included along with other tools as part of iBioSim GUI frontend, which is available for download at http://www.async.ece.utah.edu/iBioSim.

^*b*^Description of the default abstraction methods configuration for the analysis of the total and FimB-mediated ON-to-OFF switching in terms of the notation given in Figure 9.

However, such an approach to model complexity reduction could also lead to a further problem: while most abstractions used in the analysis of biomolecular networks have traditionally been implemented manually and on the mechanism-by-mechanism basis, doing so accurately in a general biological systems setting becomes tedious and time-consuming. The resulting model translation and transformation errors also tend to increase when progressively more intricate organism-scale physiological processes—from cell differentiation and tissue development to cancer, infection, host-pathogen interaction dynamics, etc.—are considered.

The strategy used here is able to substantially overcome these issues by *automating* the abstraction process via a set of algorithms developed for and implemented in the reb2sac computational tool [63],[64]. Its application has allowed us to generate abstracted representations of detailed reaction-level biological mechanisms—including genetic regulatory networks—which yield results in close correspondence with those obtained by using the underlying low-level models, while also significantly accelerating the required computations and often putting them on par with those of high-level descriptions. For instance, we were previously able to validate the overall robustness and utility of such an automated abstraction approach to biological systems analysis by using it to investigate the lysis/lysogeny developmental decision pathway in *E. coli* phage 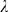 [63],[64]. The ensuing abstracted model analysis not only yields results that substantially (and in significantly less time) reproduce those elicited through the examination of the detailed system description reported earlier [65], but is further able to quantitatively investigate and similarly match experimental observations of system properties exhibited under environmental conditions that have been previously shown to cause the detailed model analysis to become so computationally demanding as to make it essentially infeasible [63],[65].

**Figure 2:**
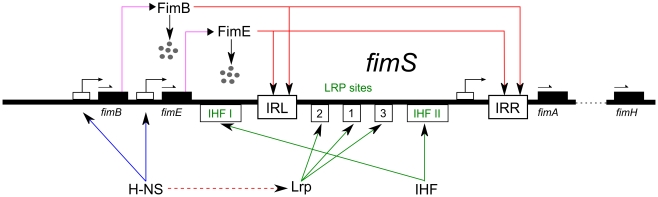
Type 1 fimbriae genetic regulatory network—the *fim* switch circuit. Structural fimbriae subunits are encoded by *fimA* and other downstream genes, which are transcribed when the *fim* switch is in the ON position (as shown here – also see Figure 1). Recombinases 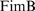 and 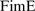 bind to 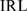/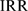 and invert the switch with different rates (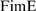 is strongly biased in the ON-to-OFF direction, while 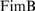 is close to fair). A small protein, 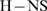, acts in a temperature-dependent manner and represses the expression of the two recombinases. 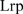 stimulates and inhibits switching based on its occupancy of three 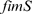 sites, while its expression is also repressed by 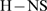. 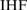 is required for any observable phase variation as it plays a structural role during switching through its ability to produce sharp bends in the DNA.

Here, we use such computational analysis aided by automated model abstraction to examine the behavior of the basic genetic regulatory network responsible for the ON/OFF switching of type 1 fimbriae expression in uropathogenic *E. coli*, Figure 2. We specifically focus on how different temperature settings quantitatively modulate the random switching of the UPEC fimbriation circuit into the transcriptionally silent *fim* mode through the corresponding ON-to-OFF inversion of *fimS*. Notably, while the behavior of most molecular processes depends on temperature, in this system global regulatory proteins 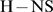 and 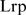 play a particularly important role in controlling switch inversion rates not only by directly effecting its internal molecular dynamics, but also by acting as sensors of certain environmental conditions that the *fim* circuit is subjected to in the physiological range—including those of a host. For instance, 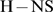 acts in a temperature-dependent manner when it binds to DNA regions containing *fimB* / *fimE* promoters and represses their expression [31],[66]. Additionally, 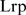 binds to three 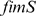 sites, which affects switching rates [50],[67],[68]. Since 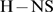 downregulates the expression of *lrp* [69],[70], 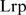 also behaves in an effectively temperature-dependent manner. Finally, it has been shown that 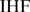 binds to 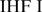/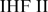 regulatory sites and is required for any observable phase variation, in part by playing a structural role in *fim* switching via its ability to introduce sharp bends into the target DNA [47],[71]. The resulting molecular interactions that involve 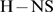, 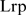, 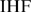, 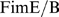 as well as the *fimS* DNA element and associated regulatory sites are what largely serves to kinetically effect the ON-to-OFF *fim* switch circuit dynamics. As the latter physiologically initiates the transition of an individual bacterium from the virulent fimbriate to the largely benign afimbriate phase and given the wide-spread emergence of antibiotic-resistant UPEC, a better understanding of such processes could benefit the development of novel clinical UPEC UTI therapies by, among other things, providing deeper insights into mechanisms potentially able to medically abrogate UPEC virulence by exploiting its internal molecular circuitry responsible for regulating the state of *fimS* in order to inhibit type 1 fimbriae expression.

Towards this end, the paper begins by considering a detailed reaction-level discrete and stochastic description of the biological molecular network controlling the *fim* switch. As discussed earlier, we then abstract this detailed representation by utilizing reb2sac, which enables us to successfully circumvent the otherwise significant computational issues involved. The accuracy of our abstracted model analysis with respect to the target system property—i.e., *the temperature dependence of the fim switch turn-off rate*—is further validated by comparing its results with those computed via the unabstracted detailed model as well as with those derived from empirical observations (where available). This, in turn, serves to explicitly demonstrate how automated model abstractions can be used to help substantially improve the speed and efficiency of biological molecular systems analysis, while also maintaining precision and improving interpretability of results. For instance, the abstracted representation has allowed us to better understand the general circuit-level organization of the regulatory logic behind the UPEC fimbriation switch and to identify the two key subnetworks—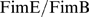 recombinase regulation and *fim* switch configuration—involved in its engineering design. Our conclusions also confirm that temperature has a major and non-trivial role in determining ON/OFF switching of fimbriae expression as well as suggest new insights into the role of 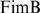 in this process and offer novel clues toward its potential translational applications in the host environment. In particular, our results indicate that—when the control circuit behavior is analyzed quantitatively across different temperatures—the primary role of 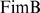 recombinase may not be to increase the total ON-to-OFF switching rate, but rather to reduce it by down-regulating the rate of switching mediated by the competing recombinase 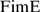. That is, down-regulation of 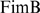 not only reduces the OFF-to-ON switching, but also serves to increase the ON-to-OFF rate in a temperature-sensitive manner, which indicates that this mechanism may provide a powerful regulatory tool for suppressing the fimbriate UPEC phase. Finally, as our analysis implies that the described effect is strongest and the switching rate is most sensitive to the corresponding mode of control in the physiological temperature range of the host environment, it may serve to potentially help identify new biomedical targets in the UPEC molecular virulence circuitry.

## Results

### Detailed Model

**Figure 3:**
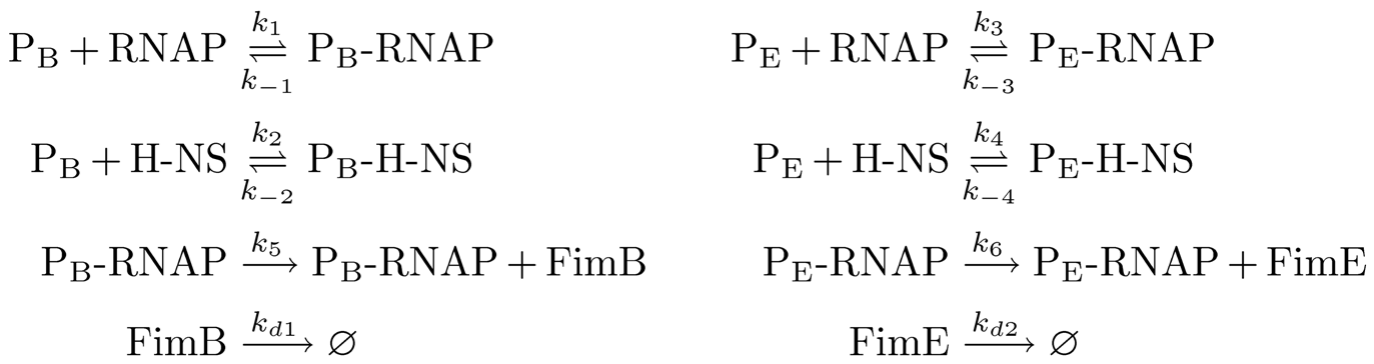
Detailed model subnetwork of FimB and FimE regulation. Here, 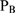 is the promoter for *fimB* and 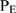 is the promoter for *fimE* . Each 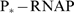 represents a transcriptionally active configuration, while 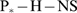 corresponds to the transcriptionally silent complex.

**Figure 4:**
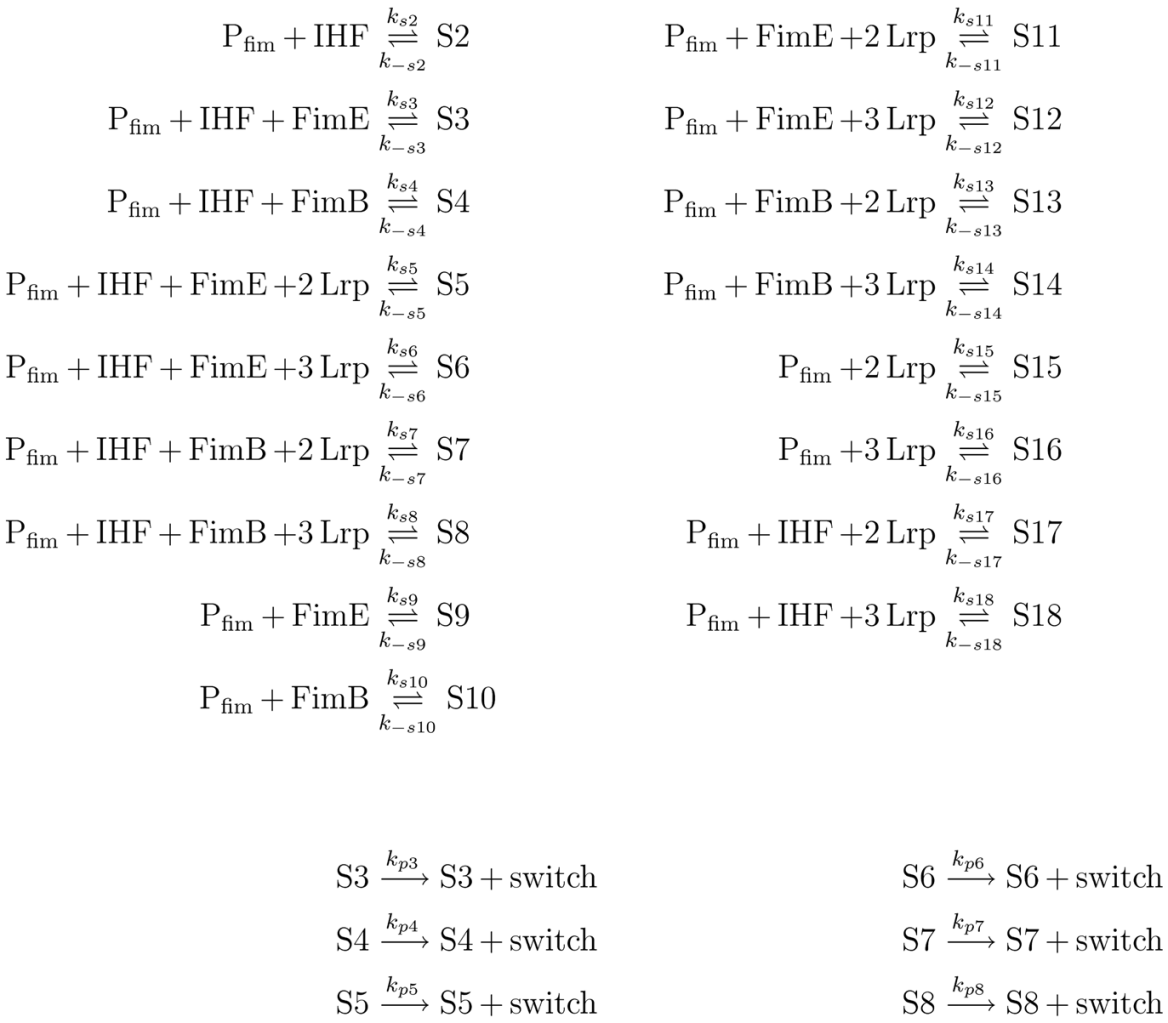
Detailed *fim* switch configuration model. Here, 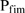 abstracts the free form of the regulatory protein binding sites in *fimS*. Complex species 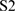 through 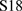 represent the various states of the *fimS* DNA element given in Table 6. An abstracted species, *switch*, captures the switching events.

Based on the regulatory network diagrammed in Figure 2, we have developed a molecular kinetic reaction-level description of *E. coli* fimbriation switch system, which has resulted in a *detailed model* of the *fim* circuit that comprises 52 reactions and 31 species (Figures 3 and 4). This model is then used to, among other things, quantitatively analyze the effects of temperature on both the total and 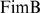-mediated ON-to-OFF *fim* switching probabilities over one cell generation. In particular, starting with the switch in the ON position at various temperature settings—i.e., 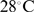, 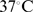, and 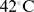
—where the corresponding empirical observations were available (see Methods and Text S1), the detailed model was simulated 100,000 times by using our implementation of Gillespie's Stochastic Simulation Algorithm (SSA). The ensuing switching behavior of the *fim* circuit was found to be both qualitatively and quantitatively consistent with that obtained via empirical observations [30] (see Table 2). However, computational demands presented by these detailed model simulations were significant, requiring over 30 hours on a 3GHz Pentium 4 with 1GB of memory (Table 3).

**Table 2:** Comparison of ON-to-OFF switching probability estimates in minimal medium.

	Probability per cell per generation ( 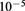 ):		
	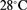	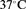	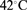
Empirical results^a^			
Wild-type	7,000	1,800	600
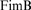 -only	69 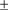 26	110 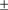 24	34 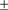 28
Detailed model^b^			
Wild-type	7,298 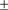 161	2,012 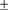 87	673 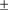 51
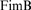 -only	67 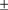 16	93 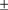 19	59 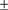 15
Abstracted model^b^			
Wild-type	7,260 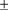 80	2,003 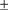 43	615 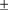 24
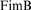 -only	77 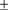 9	99 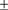 10	46 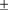 7

^*a*^Based on experimental observations reported in [30].

^*b*^Error bars correspond to 95% confidence interval calculated using the binomial distribution with 100,000 samples for the detailed model and 400,000 samples for the abstracted model.

**Table 3:** Simulation time comparison between detailed and abstracted models.

	Simulation time^a^ (hours)					
	Wild-type		*fimB* knock-out^d^		*fimB* overexpressed*^e^*	
Model	Partial^b^	All^c^	Partial	All	Partial	All
Detailed	28.5	N/A	17.1	N/A	28.8	N/A
Abstracted	1.5	2.85	0.67	1.17	2.38	4.57

^*a*^Computational time for 100,000 stochastic simulation runs as well as model abstraction when applicable for each temperature point on a single PC.

^*b*^Temperature points at 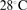, 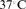, and 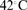.

^*c*^Temperature points at 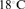, 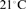, 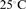, 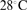, 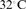, 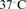, 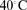, 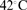, 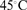, and 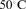.

^*d*^System with no ON-to-OFF 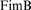 activity.

^*e*^ 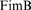 overproduction by a factor of 2 compared to wild-type.

### Abstracted Model

**Figure 5:**
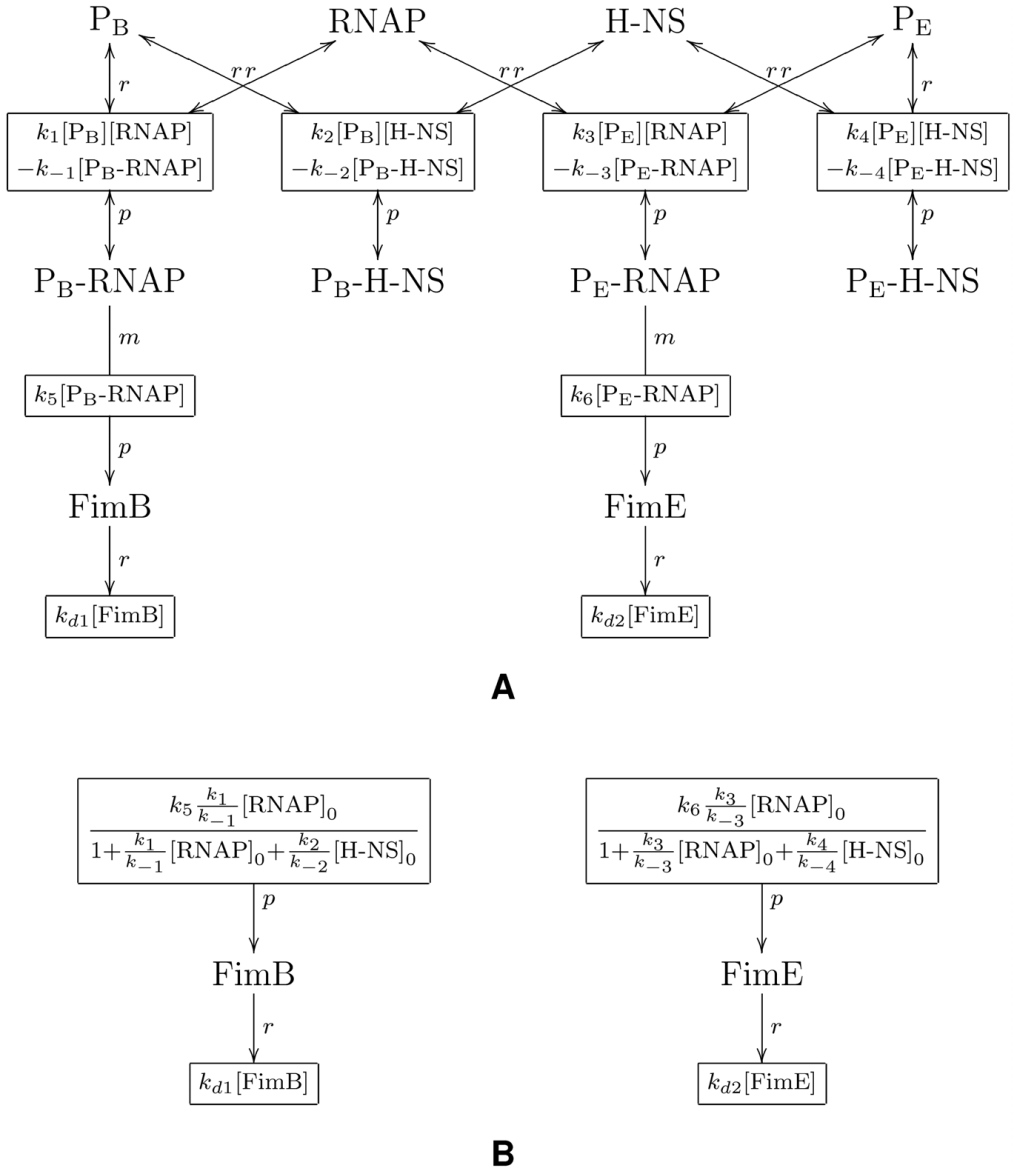
Graph-based model representation of FimB and FimE regulation subnetwork. A reaction connected to a species with a double arrow designates a reversible reaction. Species connected to a reaction with letters, *r*, *p*, or *m* corresponds to a reactant, a product, or a modifier for that reaction – respectively – as defined in the SBML standard [156]. A mathematical expression inside a reaction node provides the kinetic reaction rate function for that reaction. (A) Detailed model; and (B) Abstracted model.

**Figure 6:**
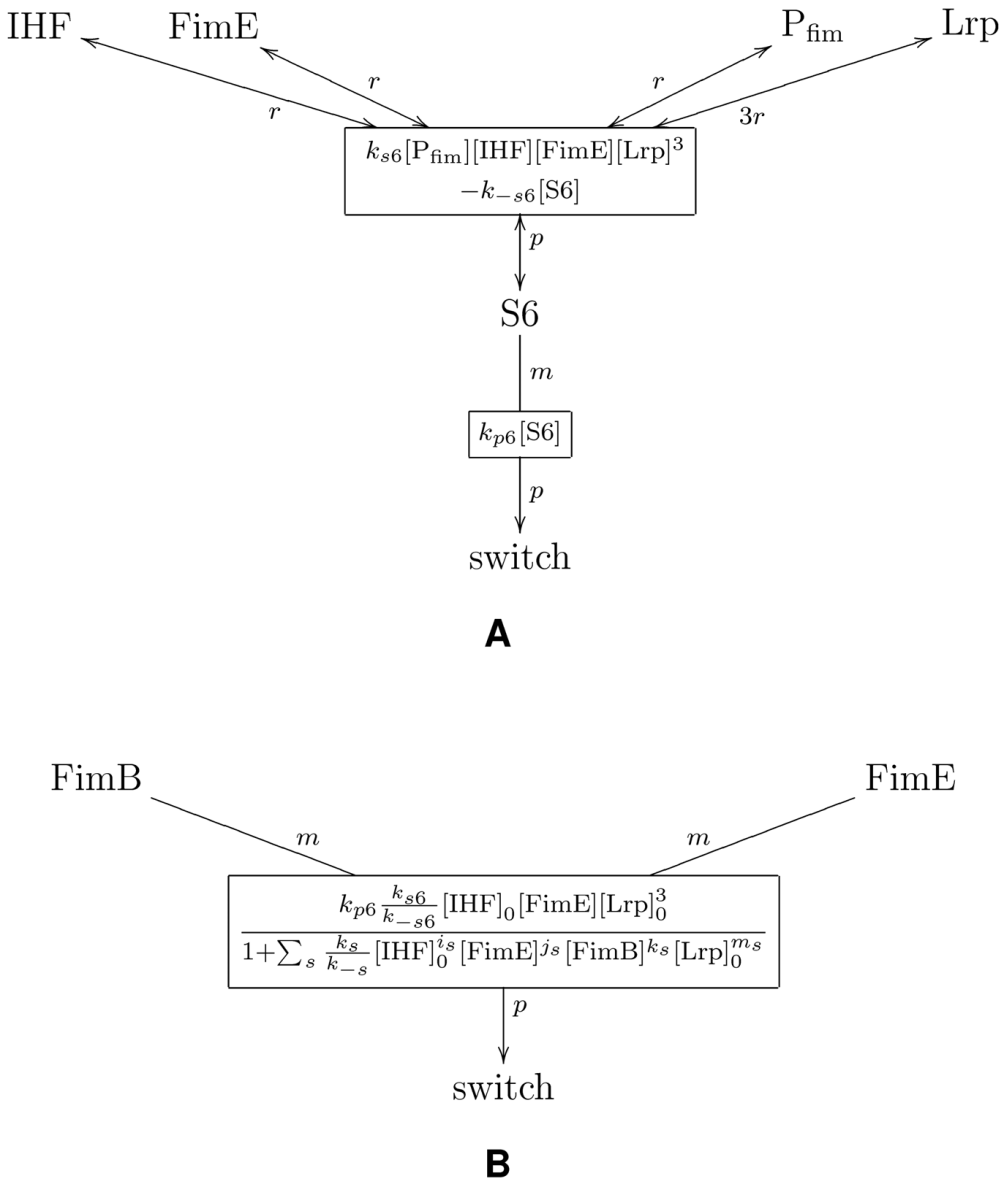
Reaction scheme for *fim* switch ON-to-OFF inversion through state 6. In this state, 1 molecule of 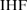, 1 molecule of 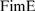, and 3 molecules of 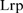 occupy available binding sites in the switch DNA region—leading to the corresponding switching event. (A) Detailed model; and (B) Abstracted model. (See Text S1 for further detail.)

After applying reb2sac automatic abstraction engine with the switch state as the target quantity of interest, the detailed model is transformed into an *abstracted model* with 10 reactions and 3 species (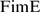, 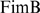, and a conglomerate non-linear stochastic switch – see Figures 5 and 6 as well as Methods for further detail). In order to compare the abstracted and detailed models, we have performed numerical simulations to compute the wild-type and 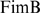-mediated ON-to-OFF switching probabilities for one cell generation in minimal medium using the same simulator. The results of the abstracted analysis are found to be in close agreement with those obtained using the detailed model and substantially match the empirical observations (see Table 2). However, computational gains from the model abstraction are significant. The abstracted model simulation of 100,000 runs takes less than 2 hours on a 3GHz Pentium 4 with 1GB of memory, which is a speed-up of about 16 times compared with the runtime of detailed model simulations (Table 3).

### Modular Organization of the *fim* Switch Circuit

In addition to allowing for accurate kinetic simulation of circuit-level dynamics, the reaction-level description of biological networks is often useful in helping to reveal their broader structural and functional features, including the innate modular architecture of *E. coli* fimbriation switch design considered here. Specifically, graph-level analysis carried out as part of the detailed model abstraction process has naturally led us to separate out and identify its two major constitutive subnetworks. These turn out to correspond to the two principal functional units of the *fim* switch circuit: the module effecting production-degradation of 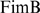 and 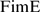; and the module responsible for the configuration dynamics of the *fimS* element itself (e.g., Figures 5 and 6). Such a view of the internal *fim* switch circuit organization both makes its logic easier and more intuitive to understand as well as simplifies and provides further basis that serves to facilitate subsequent steps involved in the model abstraction process.

### Quantitative Analysis of *fim* Circuit Switch Temperature Control via the Abstracted Model

**Figure 7:**
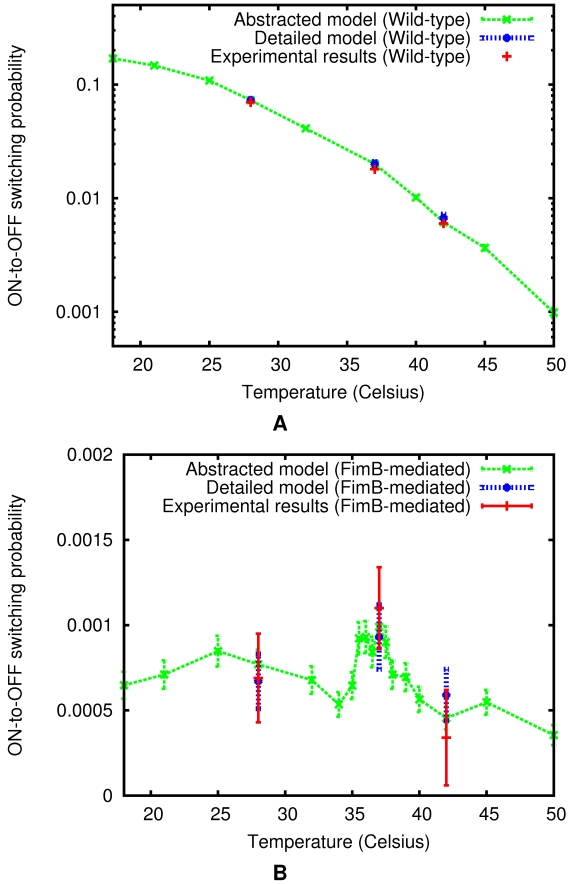
Regulation of the ON-to-OFF *fim* circuit switching probability via temperature control. The detailed model was used to evaluate ON-to-OFF switching probabilities over one cell generation at the three temperature points (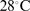, 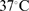, and 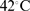), where experimental measurements had been made previously [30]. Calculations were repeated using the abstracted model at these and seven additional temperature points (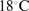, 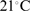, 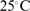, 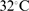, 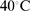, 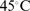, and 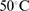) – all in minimal medium. Here, (A) Wild-type (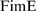 and 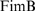) ON-to-OFF switching probability per cell per generation is plotted versus temperature; and (B) Same, but for 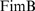-only mediated switching, where further points (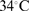, 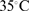, 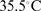, 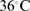, 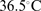, 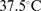, 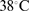, and 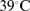) were added to increase resolution around the physiological temperature peak. (Error bars in (A) and (B) indicate 95% confidence interval.)

By systematically refining our understanding of the underlying organization logic and improving required computational times, our approach further enhances the ability of *in silico* analysis to accurately explore various environmental as well as internal conditions and parameter regions of biological systems. This may be particularly useful when certain settings can be deemed physiologically important, yet are not easily amenable to or simply do not presently have sufficient number of experimental measurements available; and which lead to dynamics that are too complex or involve species too numerous to be productively investigated directly at the detailed molecular interaction network level. For example, in the case of the *fimS* inversion control circuit, probabilities of ON-to-OFF switching at various temperature points (including those outside of the experimental range) can be effectively and efficiently estimated by using the described model abstraction methods. Here, Figure 7 shows both wild-type and 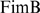-only mediated ON-to-OFF switching probabilities computed via the abstracted *fim* switch model at – respectively – 7 and 15 additional temperature points, where experimental data are not available (also see Table 2).

Notably, these results not only reaffirm earlier coarser-grained empirical observations of wild-type and 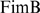-only mediated ON-to-OFF *fim* circuit switching frequency dependence on temperature [23],[30], but also offer the finer-grained resolution capable—as discussed below in more detail—of providing further insights into this relationship. In particular, while our analysis supports the prior suggestion that the wild-type *fim* ON-to-OFF rate is overall a decreasing function of temperature that varies by nearly two orders of magnitude in the physiological range, it also appears to indicate that this dependence has a supra-exponential component as well, Figure 7A. Furthermore, when the abstracted model is used to increase the resolution of FimB-mediated switching frequency dependence on temperature, it shows that UPEC may have evolved toward a tightly optimized type 1 fimbriae virulence factor expression control that is designed to sense and differentially respond based on whether the host temperature is within the normal physiological range of 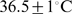 or if it is elevated/lowered instead. Whereas the circuit 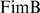-mediated ON-to-OFF rate appears to be maintained at a relatively elevated but stable level across the entire normal temperature range—it looks to be significantly suppressed immediately outside of this characteristic band, Figure 7B, which may have notable implications for the persistence of the pathogenic UPEC phase and ensuing UTIs (see Discussion).

### Role of FimB in the Temperature Control of ON-to-OFF *fim* Circuit Switching

Since the 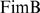-mediated switching probability can be orders of magnitude smaller than the wild type ON-to-OFF switching probability (Table 2), the effect of 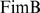 on the temperature control of the fimbriation circuit shutdown rate may also appear minimal. It is, furthermore, not immediately clear why 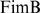-mediated switching needs to be exquisitely bidirectional rather than simply OFF-to-ON, given that 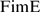 essentially only promotes ON-to-OFF switching and completely dominates the 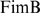 rate in this direction. While various theories have been proposed to explain this feature of the fimbriation regulatory network design (see Discussion), we wanted to generate a quantitative hypothesis regarding the role of 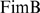 in the temperature control of the *fim* ON-to-OFF circuit switching by using computational analysis methods to perturb the underlying molecular interaction-level network properties and to then explore the behavior of any resulting fimbriation mutants. To do this, we have modified the original *fim* switch inversion system *in silico* and generated several detailed mutant models—two of which proved to be of particular interest. One represents a mutant, where *fimB* has been placed under the control of a strong promoter that leads to 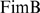 overproduction by a factor of two relative to wild-type. The other describes a mutant, such as a knockout or an amino acid substitution, where 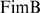 protein has been rendered nonfunctional in the present context by losing its ON-to-OFF switch-mediating activity. Both mutant models were abstracted using reb2sac and simulated.

**Figure 8:**
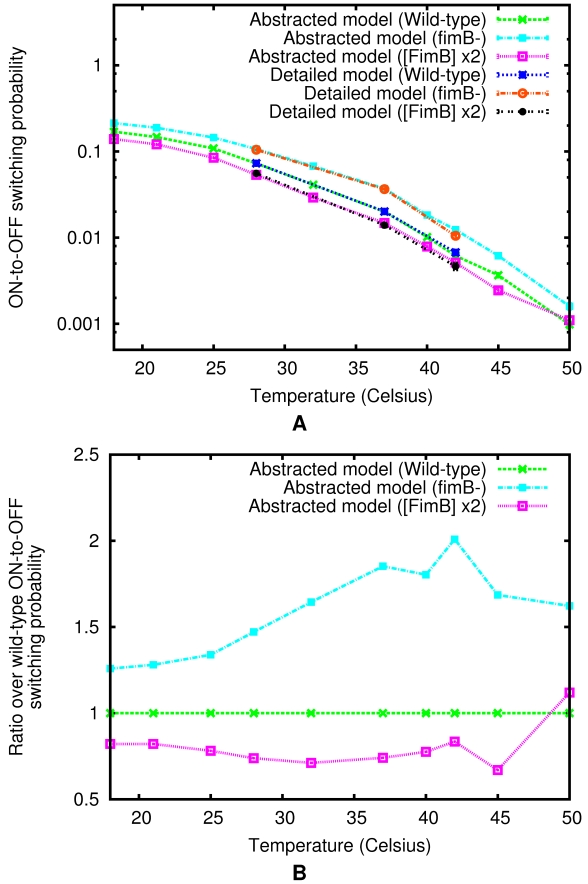
Role of FimB in the temperature control mechanism of the total ON-to-OFF *fim* switching probability. The total ON-to-OFF switching probability of two *in silico* generated mutants: one—overproducing 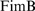 (at twice the wild-type level), and the other—a 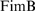 knockout (no ON-to-OFF 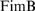 activity). These are compared with the wild-type system behavior using their respective abstracted models at the same 10 temperature points (see Figure 7A). Here, (A) The total ON-to-OFF switching probability per cell per generation in minimal medium is plotted versus temperature. For numerical comparison, each case also includes three points computed directly via the detailed model. (Error bars indicate 95% confidence interval); and (B) The ratio of the total ON-to-OFF switching probability in each of the mutants to the total ON-to-OFF switching probability of the wild-type is plotted versus temperature.

Comparing the elucidated mutant and wild-type behaviors at the same 10 temperature points considered earlier (e.g., Figure 7A) now allows us to quantitatively characterize the dependence of this *fim* switch circuit temperature control on the level of 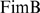 activity in the cell. As illustrated in Figure 8A, the total ON-to-OFF switching probability generally decreases inversely with 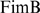 levels across all temperatures. That is, in the physiological range, the total ON-to-OFF switching probabilities in the *fimB* ^−^ mutant are higher than those in the wild-type, which are—in turn—higher than those in the mutant where 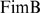 is overexpressed. Notably, this not only suggests that the 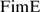-mediated shutdown of fimbriae expression is efficiently down-regulated by 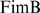, but that—as shown in Figure 8B—this effect is strongest in the 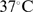 to 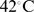 temperature range, where the total ON-to-OFF switching probability of the *fimB* ^−^ mutant can be over two times higher than that of the wild-type and nearly three times that of the overexpressing mutant. Physiologically, this implies that the presence of 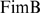 at normal or elevated levels greatly enhances the persistence of type 1-fimbriated UPEC phase. Thus, although the 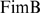-mediated *fim* switching probability is itself at least an order of magnitude lower than wild-type, 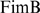 may have a key role in regulating and enhancing the control of temperature-dependent functions in the *E. coli fim* switch circuit by—among other things—also reducing the effect of 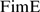-mediated ON-to-OFF *fim* switching. This serves to regulate the type 1 fimbriae-based molecular virulence mechanism and, potentially, may help increase the life-time of the pathogenic fimbriate UPEC phase. The latter result is of particular interest because the effect appears to be most pronounced in the temperature range that corresponds to the intra-host bladder environment, opening up the possibility that it may be directly relevant to UPEC-caused UTIs.

## Discussion

In recent years, rapid advances of experimental biology made it practical to study both molecular- and network-scale organization of many biological and physiological processes in much greater detail than was previously feasible. This, in turn, has made computational analysis not only possible, but also essential to any efforts aimed at understanding the increasingly intricate structures and functions of multiscale biological systems that are being uncovered through empirical means. However, this growing wealth of knowledge about *in situ* biological processes has also led to the demand for progressively more sophisticated *in silico* system models. As a result, although accurate molecular-scale biochemical descriptions could be formulated for a large number of experimentally observed systems, their complexity is rapidly exceeding our present as well as near-future computational capabilities—the issue that has become more pronounced with the emerging understanding of the ubiquitous role played by nonlinear and discrete-stochastic (“noisy”) molecular dynamics in gene regulatory, signal transduction, and other biological systems [39]. That is, while their role may often be essential in defining the various design and functional characteristics of biomolecular circuits [72]–[78]—including temperature controls [79]–[82]—the resulting introduction of multiplicative noise and the possibility of ensuing deviant effects [54], [83]–[89] can make computational analysis of such processes particularly demanding [62].

Going forward, these considerations appear to suggest that “model abstractions”—whereby, for instance, multiple biological network interactions comprising individual biomolecular mechanisms are rigorously and systematically aggregated into a few easily tractable, but functionally analogous components—will continue to become an increasingly useful tool in the general context of computational and systems biology. Importantly, model abstractions can serve not only to substantially reduce the computational requirements associated with the analysis of specific multiscale biological processes, but may also lead to identification of functional units that correspond to biologically meaningful modules or motifs (exemplified here by the two functional subnetworks of the *fim* switch circuit). The latter helps contribute additional insights into the underlying system organization and physiology as well as make their often intricate logic easier to understand.

Yet, given this growing scope and complexity of systems biological models, manual implementation of comprehensive abstractions with accuracy and efficiency becomes a challenge—creating the need for process automation. This work has demonstrated the utility of such an automated model abstraction approach by applying it to the investigation of the role of temperature in controlling the ON/OFF switch state of the *fim* genetic regulatory circuit that determines the expression of type 1 fimbriae (Figure 1), which is an essential virulence factor in uropathogenic *E. coli*—the leading cause of urinary tract infections and a major growing public health problem [1]. Insights into this fimbriation process—and, particularly, into the mechanisms that control its shutdown—may be especially useful as the widespread proliferation of antibiotic-resistant and biofilm-forming UPEC strains continues to increase the demands for novel treatment methods. In particular, a thorough understanding of their cellular network function under a range of conditions may allow us to manipulate UPEC's internal molecular virulence circuitry through external means, thus potentially opening up new approaches to modulating their pathogenicity. One such key external regulator is temperature, which not only often acts as an indicator of UTI progression and impacts its course, but may also be amenable to meaningful control in clinical settings. Furthermore, as experimental investigation of these processes *in situ* may offer a variety of practical challenges, *in silico* approaches could be very useful in helping to identify how internal molecular virulence machinery is influenced by external temperature variations. However, even in the case of the relatively small biological circuit controlling type 1 UPEC fimbriation switch considered here (Figure 2), its functions are qualitatively affected by the inherently discrete and stochastic as well as the largely nonlinear nature of the underlying biomolecular mechanisms. This necessitates the type of biological systems analysis that is capable of accurately accounting for contributions of molecular-scale reaction-level processes, which typically makes direct *in silico* studies of such systems highly taxing and investigations of detailed fimbriation circuit switch properties challenging. Here, we were able to substantially circumvent such issues through the use of systematic model abstractions, which allowed us to convert a highly computationally demanding problem of *fim* circuit switch response to temperature variations into a relatively accessible one by relying upon the automated model abstraction methodology we have developed and implemented in the reb2sac model abstraction tool [63]. We then used this abstracted model to gain deeper insights into the dynamics of this biomedically important system, including the role of 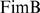 in controlling the expression shutdown rates of type 1 fimbriae virulence factor.

To do this, we have first constructed a molecular-scale reaction-based “detailed” model of the regulatory network that controls the orientation of *fimS* genomic element (Figure 2), which is responsible for ON/OFF switching of type 1 fimbriae expression. This model has allowed us to analyze—with high degree of fidelity, albeit at significant computational costs—the dynamic behavior of UPEC's discrete-stochastic genomic fimbriation circuit, including the ensuing effects of temperature on the wild-type and 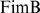-mediated ON-to-OFF switching probabilities in minimal medium, which are shown to be quantitatively consistent with those observed empirically (Table 2). We then applied our reb2sac tool to the detailed model of the *fim* switch circuit. The resulting “abstracted” model substantially reduces the complexity of the problem, enabling us to significantly increase the throughput of our *in silico* analysis (Table 3), while still maintaining accuracy when compared with the detailed model predictions and available experimental observations (Table 2). This approach has further allowed us to compute the ON-to-OFF switching probabilities at additional temperature points and to investigate the behaviors of characteristic mutants *in silico* (Figures 7 and 8).

As a result, we have been able to gain a number of insights into the internal dynamics of this clinically relevant system, including into the strong temperature dependence of putative UPEC afimbriation switching rates (e.g., Figure 7), which characterize the intrinsic dynamics that may cause individual bacteria to autonomously transition from pathogenic to benign phase. In particular, while earlier theoretical studies [90],[91] have discussed how the type 1-fimbriation level is regulated by the two recombinases, it has not been entirely clear what role (if any) 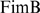 has in turning off the *fim* switch, since the ON-to-OFF rate it mediates is at least an order of magnitude lower than that enabled by 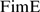. This may also seem at odds with the evolutionary selection of the remarkably fair ON/OFF 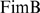 switching probabilities observed. Our analysis (which—it should be emphasized—though based on primary empirical data, is done substantially *in silico* and so needs further experimental validation) has been able to suggest a possible explanation for this ostensible contradiction by identifying a potentially key regulatory role of 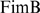 in directing UPEC afimbriation. Specifically, while the switching rate it can mediate directly remains low, 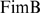 may competitively modulate the dominant 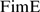-dependent switching process in excess of three-fold—thus serving to significantly lower wild-type *E. coli* ON-to-OFF switching rates in the host environment. This process can help to further prolong or abridge the persistence of the fimbriate phase in individual bacteria, which may be crucial for UPEC survival when colonizing bladder and invading urothelium, while trying to escape immune system responses and effects of antibiotic treatments, Figure 8. Furthermore, this 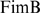-based regulation mechanism may be more robust against small perturbations in 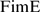 level than a simpler *fim* switch inversion control, which could be of importance in a highly variable and often rapidly fluctuating environment of the urinary tract.

While the extent to which these innate mechanisms are able to curtail or enhance virulence of UPEC *in situ* could be affected by the various aspects of complex host-pathogen interactions noted previously, it may be worth considering that to date much of the discussion has been framed in the context of such immune response processes as cytokine production, resulting inflammation, and potential subsequent exfoliation of infected bladder epithelial cells that generally lead to the increase in local tissue temperature [27],[52],[92],[93]. However, our results support a further understanding of UPEC adaptation to this aspect of host immune response. Although 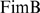-mediated fimbriae expression shutdown rate appears elevated but largely insensitive to temperature in the normal range of a host, as temperature increases further—both 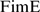 and 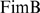 ON-to-OFF switching rates are lowered, while *E. coli*'s ability to control this process through variations in 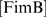 becomes optimized. That is, as UTI triggers the onset of an inflammatory response, the resulting increase in temperature tends not only to lock this UPEC control circuit in the pathogenic fimbriate phase, but also to transiently maximize switch sensitivity towards regulation by 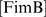 at several degrees above normal—a range consistent with the corresponding host environment. The potential existence of such sensitized “pathogenic phase lock” (PPL) mechanism and its ensuing effects on UPEC virulence could have direct bearing on some of the clinical challenges in treating UTIs discussed earlier, since many of these characteristics are thought to be associated with type 1 fimbriae-dependent biofilm and IBC formation [15],[16]. The latter structures have been shown to provide persistent pathogen reservoirs in bladder tissue and/or on abiotic surfaces (e.g., those of medical implants, such as catheters) even in cases when antibiotic treatments can effectively sterilize urine [92]. Still, currently recommended treatment strategies include ongoing prophylactic daily or weekly antibiotic therapy in cases of recurrent UTIs (defined as more than 2 episodes in 12 months), even though studies have shown no long-term reduction of UTI recurrence in such patients after prophylaxis cessation as compared with those in placebo groups [94]. Given further risks of various potential side effects—which can range from moderate to severe—and development of drug resistance as well as a number of other undesirable consequences, including growing epidemiological and public health implications [1],[21],[94], presently available basic antibiotics-based UTI treatment strategies cannot be considered satisfactory. In fact, it has been strongly suggested that from a clinical perspective the use of traditional antibiotic therapies cannot be successful against biofilm/IBC-forming bacteria and that other treatment modes, particularly those that target biofilm/IBC/fimbriation-specific processes, need to be developed [95],[96]. Thus, inference of type 1 fimbriae expression regulation circuit logic and elucidation of external intervention strategies able to influence or interfere with its internal dynamics, including via mechanisms that utilize controlled temperature variation to induce PPL relief and subsequent *fim* switch shutdown as discussed here, could offer promising potential for contributing further understanding towards the development of novel remedial approaches.

Historically, many such original medicinal and other therapeutic methods have had their genesis in traditional or domestic practices [97]—a pattern that has been recently seen to accelerate because of, among other things, growing synergies between Western and Asian medical systems that have already resulted in such notable pharmacological and synthetic biological successes as *ephedrine* and *artemisinin*—with more on the way [98],[99]. For instance, while a relatively prolonged exposure to cold has been generally associated with the increased incidence of UTIs [100],[101], a number of complementary therapies have been based around the practice of keeping genitourinary tract area cool or even briefly exposing it to low temperatures as beneficial for the prevention and treatment of various pathological processes, including microbial infections [102],[103]. Yet, while the ongoing research into the effects of cold exposure on differential activation/repression of various adaptive and innate immune system components has now begun to suggest underlying cellular and molecular biological basis for these phenomena observed in clinical applications, their underlying modes of action on the whole remain poorly understood [104],[105]. In this context, the results discussed here provide an example of the quantitative insight that multiscale reaction-based computational modeling brings to such complex processes. Specifically, based on the implications of our study for utilizing alternative temperature-driven approaches in targeting the dependence of UPEC virulence mechanisms on type 1 fimbriae expression—rather than relying solely on antibiotic or other biochemical means—two mechanisms may merit further attention. On the one hand, as host response to UTI includes tissue inflammation and a corresponding local or systemic *increase* in temperature, our analysis indicates that the adaptive feedback strategy evolved by UPEC tends to bring about PPL conditions, whereby ON-to-OFF type 1 fimbriation circuit switch may become maximally sensitized to 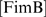. Combined with its central role in mediating the OFF-to-ON switching [47], this implies that lowering 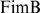 activity may lead to a reciprocal decrease in the fraction of virulent fimbriate UPEC phase and subsequent reduction in the associated pathogen load—making the corresponding persistent UTIs more amenable to host immune mechanisms and, potentially, increasing the efficacy of existing medical treatments. However, given the challenges of developing and delivering the required inhibitors as well as further obstacles presented by IBC formation inside epithelial cells, it may not be immediately clear how direct variation of UPEC 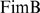 activity could be meaningfully achieved *in vivo*. On the other hand, our conclusions also support the notion that *decreasing* the temperature of UPEC environment may increase shutdown rates of type 1 fimbriation circuit switch (including by indirectly lowering 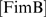), thus potentially leading to the up-regulation of afimbriation rates in individual bacteria. This would tend to suppress UPEC pathogenicity by reducing their capability for attaching to and invading urothelial cells as well as by interfering with biofilm/IBC formation and maintenance, which may be expected to decrease their capacity for subsequent re-infection. As in this case only local temperature variations—including those directed by cool/warm intravesical media or such catheter and other device instillation—are principally required in order to elicit the indicated physiological response, the conditions necessary to influence UPEC fimbriation switching in this manner may be practically attainable in biomedical and clinical applications.

It is important to note, however, that this merely suggests the possibility and does not engender any further assessment of potential efficacy such therapies may have in clinical UTI settings. The latter requires a more extensive follow on investigation—particularly in view of additional host-pathogen interaction dynamics, the multicellular nature of the system and commensurably greater complexity of intra-/inter-cellular networks it comprises, the epidemiology of autoinfection processes involved in promoting UTIs from and diversity of the endogenous bacterial flora, etc. as well as any associated difficulties in developing detailed models of the intra-host pathogen environment. Such challenges are often due to our understanding of biomolecular functions involved being insufficiently detailed and/or tissue-specific processes adding further layers of complexity to the overall infection dynamics. For instance, while this work has been able to use modeling and computational analysis in order to explore certain aspects of type 1 fimbriae switch control, the latter are primarily relevant to lower urinary tract infections. In contrast, upper UTIs are predominantly promulgated by P fimbriae—a distinct UPEC adhesive factor, which is regulated by significantly different biomolecular circuitry (see [106],[107] for detailed modeling of the corresponding *pap* switch) that leads to its own mode of thermoregulation [108]. Still, recent experimental results—from those cited earlier with respect to UPEC and host immune system, to the discovery of TRP channel family of cold and hot sensors in human genitourinary tract [109]—have provided strong evidence that temperature and its variations can have major systemic influence on healthy functions as well as various pathological developments in the urinary tract and surrounding tissues. In fact, basic intravesical cooling or warming with media of desired temperature or via chemical agonists, such as menthol/icilin or capsaicin/resiniferatoxin – respectively, has had a long history of being used to induce nerve desensitization, bladder cooling reflex, and other physiological mechanisms in therapeutic applications ranging from treating patients with detrusor overactivity, bladder pain, and urothelium irritation to diagnosing various urinary tract and neurologic disorders [109]–[111]. This not only directly indicates that patient urinary tract temperature could be practically and therapeutically manipulated in clinical applications, but—as TRP sensors appear specific to animals and fungi [112]—also suggests that thermal regulation of human physiological response processes may be actively effected in a manner that by-and-large does not directly impinge upon prokaryotic pathogens. Conversely, with better empirical understanding and computational modeling of the underlying biological circuits, the same mechanism may allow us to substantively offset the effect on the host of moderate temperature changes by applying compensatory chemical stimuli to appropriate TRP channels and modulating their ensuing activity up to desensitization. This, in turn, opens up the possibility that externally controlled temperature variations may be guided by quantitative systems analysis to specifically target and manipulate the internal dynamics of bacterial or other pathogenic processes *in sutu*, causing them to either become innately less virulent—for example, as has been discussed here in the context of UPEC fimbriation circuit switching—or making them more susceptible to the immune response as well as antibiotic and other treatments, thus potentially contributing to the ongoing enhancement of existing and the development of novel therapeutic applications.

Taken together, these results broadly serve to further demonstrate the potential utility of computational and systems biological approaches as we are beginning to understand and control many physiological processes in disease and development at the inter-/intra-cellular network and circuit levels [113]–[118], thus enabling greater insights and providing more effective solutions to associated clinical and public health problems. They also highlight the benefits of model abstractions and the need for process automation as tools of *in silico* biological systems analysis, including their ability to significantly increase the efficiency with which practical multiscale biomolecular and biomedical problems may be addressed *in situ*. In fact—while this may be directly noted by considering just how much longer it takes to simulate a detailed network model, or how tedious a manual implementation of all constitutive abstractions can be, or significant simplifications in functional logic the corresponding process modularization may be able to achieve—what ultimately makes the automated model abstraction approach compelling is the eventual consideration of how relatively simple the *E. coli* type 1 fimbriation switch circuit and its temperature controls appear to be as compared to the complexity of many other biological and biomedical processes we may be expected to face in the context of systems and computational biology now or in the near future.

## Methods

Previous works by Wolf & Arkin, Blomfeld et al., and others have helped elucidate and ascertain the importance of discrete and stochastic mechanisms in the *fim* system dynamics [23],[30],[45],[47],[71],[90],[91]. For example, it has been shown that *fimS* inversions are digital (ON/OFF) events that are randomly promoted by 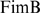 or 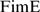 binding to discrete IRL/IRR sites and regulated by the corresponding 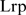 or 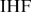 occupancies of *cis*-regulatory genomic elements, which are present in low integer counts. Under these conditions, biomolecular systems can manifest emergent and unintuitive behaviors that may greatly deviate from the predictions of macroscopic continuous and deterministic classical chemical kinetics (CCK – also referred to as reaction rate equations or mass-action kinetics) [54]. Therefore, accurate analysis of the *fim* switch circuit requires the use of a mesoscopic discrete and stochastic process description based on the chemical master equation (CME) [54],[56],[58],[59],[119],[120].

This approach considers the behavior of biomolecular systems at the individual reaction level by exactly tracking the time-evolution of the discrete number probability distribution for all molecular species present in the system and by correspondingly treating each reaction as a separate random event. An intuitive basis for the (forward) CME can be described as follows: given 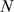 species at time 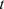 with the number of molecules 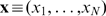 each, which are interacting through 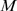 irreversible chemical reactions 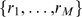 with stoichiometric vectors 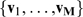 inside a well-stirred tank of constant volume and in thermal equilibrium at constant temperature—the probability that this system is found in the molecular number state 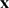 at time 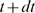 can be simply expressed as the sum of probabilities that: (*i*) the system is in the same state at time 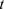 and does not undergo any transitions; and (*ii*) the probability that it is in a different state at time 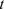 and transitions into 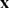 during 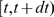. Then, under the Markovian assumption:
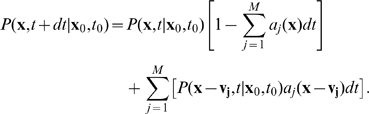
(1)with 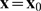 at 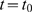 and 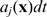
—the probability that during 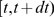 the system in state 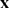 undergoes reaction 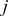
—where 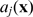 is called the *propensity function* and it is further assumed that 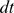 is chosen small-enough that almost surely only one reaction occurs during this time increment.

Taking the limit 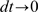 and rearranging Equation 1 gives the expression describing the temporal evolution of 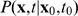:
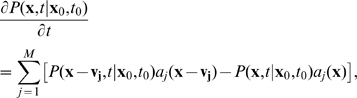
(2)which is the CME form most often used in biological applications [55]–[57],[119].

### Numerical Simulations

Unfortunately, solving the CME exactly for most biologically, physiologically, or clinically meaningful systems is typically not feasible either analytically or numerically due to the intrinsic complexity of its differential-difference form. To address this problem, a number of alternative methods—focusing on approximate analytical solutions, general computational techniques, and a range of specific applications—have been developed [62], [121]–[126]. In practice, many of these methods either derive from or have their genesis in the Gillespie's Algorithm (SSA), which enables one to gain insight into possible temporal behaviors of the system by specifying how its sample paths can be exactly drawn from the CME-described probability distribution [62],[127],[128].

Our numerical simulations approach is based on the SSA and, specifically, is implemented as a streamlined version of Gillespie's Direct Method [127]. This is a kinetic Monte Carlo simulation procedure, which—given the system in state 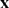 at time 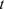
—determines per iteration: (*i*) the waiting time to the next reaction, 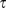, based on an exponential random variable with mean 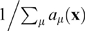; and (*ii*) the index of the next reaction, 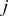, based on an integer random variable with probability 
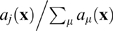
. (While the Next Reaction Method [129] is often considered to be the most efficient implementation of the SSA, recent study has discussed how the optimized version of the Direct Method generally performs better for many practical biochemical systems—largely owing to the high computational cost of maintaining extra data structures [130].) Our implementation is similar to other optimized versions of the Direct Method in the sense that it only evaluates propensity functions as necessary to minimize updates. The main difference is that our implementation does not create a dependency graph, but rather utilizes the bipartite graph structure of the reaction-based model to determine which propensity functions must be evaluated (see FimB and FimE Regulation Subnetwork section below for additional detail).

Using this implementation of the SSA in reb2sac, each simulation starts with the switch in the ON position and is run for up to one cell generation of 20 minutes as in [90]. If the switch moves to the OFF position within this time limit, the simulation is then counted as an ON-to-OFF switching event. The ON-to-OFF switching probability is calculated as the number of ON-to-OFF switching events divided by the total number of simulations with the same initial conditions. Alternatively, this could be viewed as computing the total ON-to-OFF switching probability by summing up switching events involved in all possible transition states, while the 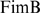-mediated events only include transitions carried out due to the binding of 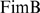
—i.e., those going through switch states S4, S7, and S8—see Figure 4.

### Detailed Fimbriation Switch Circuit Model

Our detailed switch inversion model represents a molecular reaction-scale description of the *fim* circuit (Figure 2), which generally satisfies the Markovian requirement of the SSA. (The discussion of how the individual reactions have been parameterized as well as generally identified from literature can be found below and in Text S1.) Such representations typically constitute the lowest-level (highest-resolution) description of biological systems used in most practical applications, which is one of the reasons why this model is correspondingly referred to as “detailed”.

The reaction network graph examination carried out as part of the motif recognition, data flow, system organization, and abstraction analysis has led us to identify two major modules responsible for dynamically controlling the *fimS* inversion process as well as integrating external signals provided by global regulator proteins and environmental factors, such as temperature, thus entailing a number of significant analytical and computational simplifications. These subnetworks may be broadly labeled as: (*i*) the production-degradation processes of 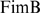 and 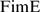; and (*ii*) the processes regulating the configuration of the *fim* switch itself.

#### FimB and FimE regulation subnetwork

As discussed earlier, FimB and FimE site-specific recombinases are essential to fimbriation circuit switching as enablers of the *fimS* inversion process. What is less immediately obvious, however, is the key role they play in receiving environmental signals, including temperature, and feeding this information into the *fim* configuration subnetwork for integration into the switch inversion decision. The temperature regulation facet of this process is effected by the substantial thermal sensitivity of the 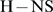-mediated *fimE* and *fimB* promoter repression. Notably, such temperature control is relatively stronger across much of the physiological regime relevant to the *fim* switch circuit operation than the effect of 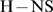
's own concentration variations due to external factors (also see Text S1).

The reaction-based description of FimB and FimE regulation subnetwork used here is given in Figure 3. However, for many applications—including our modeling and analysis tool reb2sac—a (bipartite) graph representation of biochemical networks may be more desirable [63]. In this description, species and reactions correspond to nodes connected by the respective interactions. Figure 5A provides such a graphical representation of the detailed 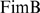 and 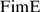 regulation model used in our analysis. Aside from its simplicity, which also aids visualization of underlying biomolecular processes, representing biochemical networks in such a graph form further offers several additional advantages for our analyses. Two major ones include: (*i*) the efficient traversal of the reaction network, which is crucial for pattern matching and subsequent model abstraction; and (*ii*) an optimized implementation of the stochastic simulation algorithm without the need for constructing additional data structures— such as dependency graphs—which minimizes the number of updates.

Table 4 provides the list of temperature-dependent rate constants and initial species concentrations involved in the 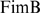 and 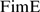 regulation process, Figure 3, across the relevant range of degrees. Table 5 lists the remaining rate constants and initial species concentrations.

**Table 4:** Temperature-dependent rate constants and parameters in the FimB and FimE regulation module.

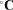	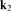 (nM^−1^s^−1^)	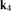 (nM^−1^s^−1^)	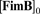 (nM)	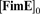 (nM)	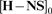 ( 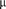 M)
18	0.001149425	0.000006964	74	199	30
21	0.001149425	0.000047619	74	188	30
25	0.001149293	0.00025	74	146	30
28	0.001133787	0.000666667	74	100	30
32	0.001132503	0.001923077	94	69	20
37	0.001	0.005524862	100	31	20
40	0.000775194	0.01	113	16	20
42	0.000588235	0.014705882	127	13	20
45	0.00034662	0.025641026	153	8	18
50	0.000133209	0.084033613	183	3	15

The values listed here are derived based on the results provided in [31],[65],[90],[132]. See Text S1 for further detail.

**Table 5:** Temperature-independent rate constants and parameters in the FimB and FimE regulation module.

Rate constant	Value	Rate constant	Value
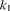	0.333333333	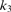	0.333333333
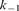	10	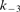	10
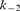	10	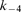	10
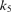	0.666666667	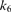	0.666666667
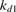	0.001625	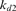	0.001625

The values listed here are derived from the results provided in [31],[65],[90],[132]. See Text S1 for further detail.

**Table 6:** Configuration of *fimS* DNA element for the ON-to-OFF switching.

State	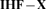 a	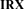 b	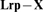 c	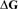 (kcal)	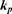 d (s^−1^)	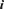 e	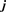 f	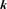 g	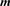 h
1	-	-	-	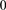	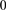	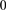	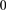	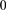	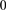
2	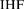	-	-	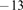	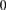	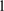	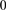	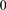	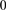
3	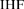	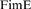	-	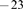	6.53e-8	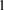	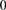	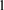	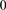
4	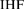	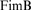	-	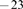	6.5e-7	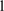	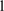	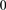	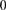
5	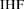	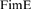	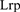	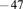	3.0e-4	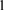	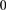	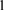	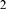
6	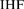	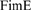	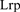	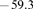	8.0e-5	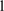	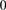	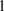	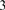
7	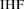	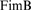	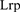	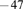	3.7e-6	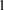	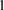	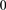	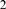
8	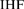	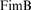	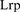	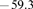	7.5e-7	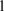	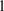	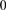	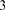
9	-	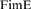	-	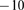	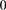	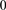	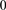	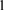	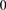
10	-	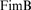	-	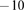	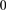	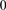	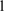	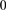	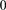
11	-	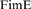	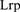	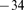	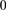	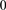	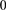	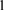	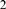
12	-	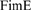	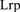	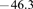	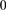	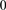	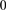	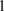	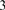
13	-	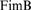	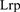	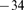	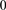	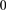	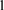	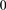	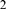
14	-	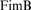	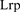	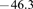	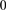	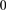	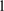	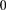	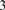
15	-	-	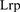	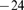	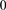	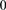	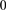	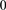	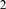
16	-	-	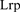	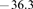	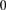	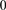	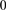	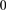	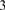
17	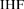	-	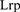	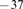	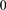	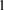	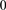	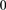	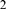
18	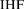	-	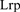	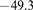	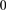	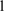	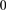	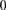	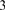

^*a*^ 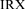 represents both IRL and IRR sites, to which the two recombinases can bind to invert the *fim* switch.

^*b*^ 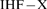 corresponds to the two 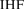 binding sites, IHF I and IHF II.

^*c*^ 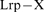 represents the three 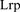 sites: Lrp-I, Lrp-II, and Lrp-III.

^*d*^ 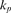 represents the switching reaction rate constant.

^*e*^ 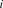 represents the number of molecules of 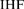 bound to the switch DNA region.

^*f*^ 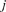 represents the number of molecules of 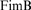 bound to the switch DNA region.

^*g*^ 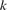 represents the number of molecules of 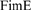 bound to the switch DNA region.

^*h*^ 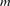 represents the number of molecules of 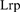 bound to the switch DNA region.

Configuration parameters are based on those for the ON state given in [90].

#### The *fim* switch configuration subnetwork

The second major subnetwork centers around binding and unbinding reactions of *fim* switch regulatory proteins, leading to ON-to-OFF phase inversions and thus involving the *fimS* invertible DNA element itself. This subnetwork is derived from the 18 configurations that the switch DNA region can be in based on the occupation of various binding sites by regulatory proteins. The reaction-level description of this module is given in Figure 4. We have been able to further quantify these processes by first reverse-engineering the underlying reactions from the equilibrium statistical thermodynamics model. That is, we have used the assumption that the regulatory molecule binding and unbinding reactions are much more rapid compared with the associated switching or gene expression rates [131]. (See Text S1 for detail.) Furthermore, this paper has taken the various types of recombination complexes (recombinasomes/invertasomes), S#, to be independent in that there is no direct interconversion between any pair of S#'s without an initial complex disassociation (see Figure 4). This is based on the understanding that the formation of a recombinasome results in DNA deformation and steric re-arrangement that prevent further binding or unbinding of other constituent molecules—such as 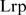
—while a recombination event has not been resolved (e.g., see [71]), thus preventing direct transitions among S#'s. (Similarly, this paper has taken subsequent complex breakdown to be complete and not partial, because the rate of switch inversion event occurrence is much slower than the kinetics of molecular binding and unbinding.)

Besides the regulatory factor binding/unbinding to/from *fimS* DNA and the 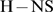-mediated repression of *fimE* / *fimB* described earlier, another main mode of temperature control in the *E. coli* fimbriation switch circuit is through its effect on the abundance of the 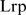 protein, Table 7. The concentration of 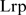 is shown to be an increasing function of temperature whereby the *lrp* expression is up-regulated as the former increases owing to the reduction in 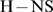-based repression [69],[90],[132].

**Table 7:** Concentration of Lrp at various temperatures.

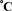	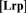 (nM)	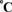	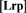 (nM)
18	2	37	5
21	2	40	11
25	2	42	20
28	2	45	45
32	3	50	130

See further discussion in Text S1.

### Model Abstractions—a Tool to Aid Quantitative Analysis of Complex Biological Systems

While SSA offers a powerful method for numerically analyzing the behavior of discrete-stochastic biomolecular interaction networks, relying on just one or several simulation runs in order to gain a general understanding of a biological system subject to stochastic decision-making, such as UPEC fimbriation ON/OFF switching, could often be misleading because—similarly to the use of CCK—randomly-simulated individual sample trajectories of the underlying stochastic process are frequently insufficient to characterize its overall probabilistic dynamics [54]. In such settings, it typically requires thousands or more simulations in order to estimate the behavior of a system with reasonable statistical confidence. Yet, because SSA needs every single reaction event to be simulated one-at-a-time, it commonly leads to very high numbers of reaction events per given time step, particularly when the system has large characteristic time-scale separations. This makes computational requirements of exact numerical discrete-stochastic analysis exceedingly demanding for most practical biological and biomedical applications. In addition, the underlying complexity of biological chemical reaction and physical interaction networks as well as their innately differential response to varied environmental conditions generally impede qualitative interpretation of biological system organization and behavior. That is, though *detailed* reaction-level representations of biomolecular networks allow for very comprehensive descriptions of biological mechanisms, such low-level models can lead to substantial computational costs as well as may, potentially, obscure the overall system structure and dynamics. The problem could be further exacerbated by the particular choices of initial and environmental conditions that biological systems are embedded in. For example, while this paper discussed the behavior of the *fim* circuit in *E. coli* growing on minimal liquid medium, the *in situ* observed switching characteristics may be altered on rich liquid or solid medium [30]. Note that these adjustments in environmental conditions should not be expected to affect the underlying molecular reaction network structure of individual bacteria (since such variations do not determine the presence or absence of constituent elementary biomolecular interactions—only their rates), but rather lead to changes in observations due to effects ranging from heterogeneity in population dynamics among cell colonies on solid medium to input-driven modulations of various process rates comprising the circuit when switching to rich medium. Accurate analysis of the system in the former case requires application of dedicated population modeling schemes that themselves can lead to non-trivial empirical effects [35],[36],[133], thus creating further modeling complexity outside of the present scope. Similarly, in the latter case, changes in empirical settings—such as growing bacteria in a rich medium—tend to produce selective increases of some cellular process rates (e.g., those involved in metabolism/degradation or cell-division) that nevertheless leave many others unchanged. This introduces further time-scale separations into the problem, thus potentially making exact numerical analysis of discrete-stochastic circuit dynamics accessible in a minimal medium, but infeasible in a rich one [63],[64].

One approach toward addressing such challenges is the ongoing development of advanced analytical and numerical approximation methods—whether with respect to time (e.g., tau-leaping [60],[134]), state space (e.g., finite state projection [135],[136]), or other system variable—that are capable of significantly accelerating the analysis of master equation-type models to within a specified level of precision. This potentially makes feasible accurate computational analysis of molecular dynamics behind physiologically-meaningful biological networks that are otherwise too demanding for exact kinetic simulations (as, for example, is the case with bacterial systems grown in rich media or other such initial/external conditions). Thus, derivation and use of quantitatively analogous, but qualitatively and computationally simpler higher-level *abstracted* representations—which could be efficiently accomplished through systematic and, given the complexity of most biological processes, automatic application of various model approximations and simplifications—becomes essential [60], [62], [63], [134], [135], [137]–[142].

In practice, this could be done by utilizing a variety of techniques. For example, *rapid-equilibrium* and/or *quasi-steady-state* approximations [143]–[145] are often used to eliminate the various intermediates without significantly compromising our quantitative understanding of the overall system logic and functionality. Other methods may include: *irrelevant node elimination*, which removes species and reactions irrelevant with respect to the species of interest by statically analyzing the structure of the model; *modifier constant propagation*, which replaces a species-state variable in kinetic laws with the corresponding initial value and removes that species if that variable is statically known to be fixed; *stoichiometry amplification*, which amplifies stoichiometries and reduces the values of propensity functions—making the system and time advancement per reaction larger; and a number of additional approaches—many of which have been implemented in our reb2sac tool (see Table 1) [63],[64],[138]. The key principle behind most of these techniques could be summarized as identifying and abstracting away various redundant or largely irrelevant variables, whose dynamics do not independently influence the behavior of the system under a particular set of conditions—or, equivalently, finding a reduced set of parameters containing sufficient information to indentify system states and transitions between them. Since in the probabilistic context all information about a system is contained within its PDF, this could be viewed as finding a minimal subset of variables or their combinations that span the range of most likely/relevant states and elucidating abstracted laws governing their dynamics from those of the detailed description. (Various methods are available for quantifying the amount of probability distribution thus captured. For instance, information entropy and mutual information could be utilized for identifying the effective complexity of processes involved as well as further used to solve the inverse problem of elucidating system structure based on observations of state occupancies, such as inferring biomolecular network organization from individual species numbers [113], [146]–[149].) Alternatively, having identified the region of state space where most of the system's probability is localized, one may seek to restrict the problem to this lower-dimensional subspace, so as to obtain the corresponding reductions in problem complexity or otherwise coarse-grain its resolution when away from most relevant states and timescales. These approaches can be particularly fruitful when applied to biological molecular systems, whose probability distributions can be described by the CME. The latter offers a well-defined analytical structure for rigorously developing such approximations—which has led to several novel methods being proposed and applied in recent years [136], [137], [150]–[154]. (For example, it has been shown that master equations for switching systems can often be projected to much smaller dimensions with little loss in their accuracy [155].) Notably, since these methods are generally based on deep theoretical understanding of the underlying molecular chemical kinetics and reaction network graph analysis, the resulting abstracted models—such as those generated by reb2sac—on balance could be commensurably expected to accurately capture the overall biological system behaviors as well as to provide rigorous quantification of any potential divergences between the abstracted and detailed descriptions.

### Automated Model Abstraction

**Figure 9:**
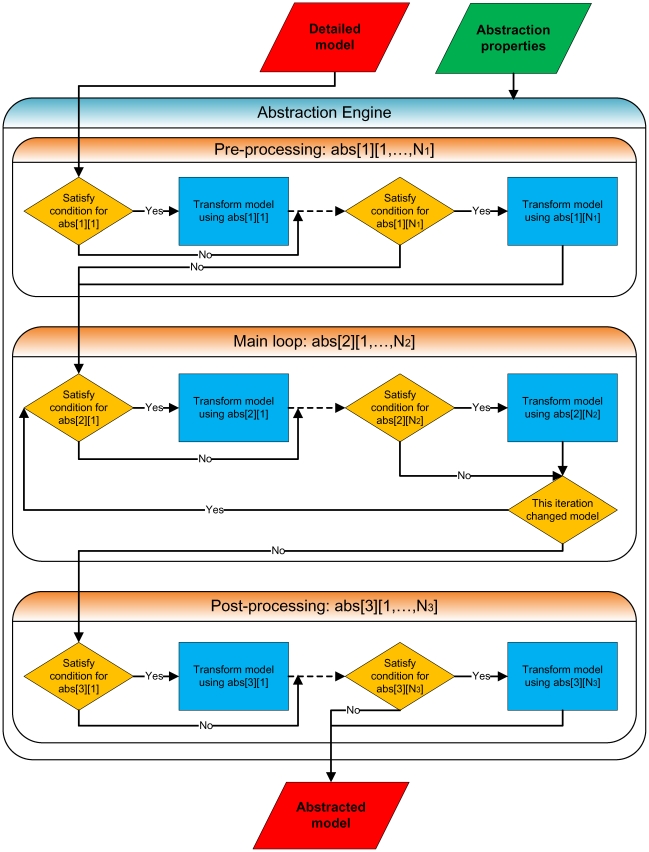
High-level workflow of reb2sac automated model abstraction engine. The engine automatically generates an abstracted model by taking as inputs a detailed interaction-based model and, optionally, various targets and tolerances that can help set and adjust the level of abstraction. A list of available abstraction methods (which include graph-theoretical interaction-network analysis tools, dynamic reaction-level approximations, etc.) is sequentially checked and, if appropriate, the method is applied to the original detailed model—transforming it accordingly. This procedure is then repeated using the next method until the list of available methods is exhausted. (See Refs. [63],[64] for further description and explanation.)

Although many approximation and abstraction approaches have been in wide use individually, their traditionally manual implementation grows to be increasingly more tedious and demanding as multiple methods are collectively applied to progressively larger biological systems. This problem is becoming even more acute as advances in systems biology continue to drive rapid increases in the typical size of analyzed networks, eventually rendering them intractable to interaction-level investigation and potentially leading to significant errors in large model transformations required to generate accurate intermediate-level abstractions. Our approach alleviates these problems by using a set of novel and existing algorithms—implemented in the reb2sac abstraction and analysis tool—to automatically survey and test biological networks for patterns and characteristics amenable to various complexity reduction techniques at the given level of accuracy for some specified “target” system property of interest [63],[64]. Among other things, this allows reb2sac to systematically scan through intermediate abstraction levels, to then automatically identify and implement appropriate approximation methods according to user preferences, and—by setting precision thresholds—to ultimately generate abstracted system models optimized for computational efficiency versus accuracy as desired. A high-level flow chart of our automated abstraction methodology is given in Figure 9. Note that the outlined analysis framework is overall quite generic and so could be used not only to generate model abstractions of gene regulatory networks, but also of other biochemical/biophysical reaction systems—including signal transduction pathways, metabolic networks, and other epigenetic processes.

Specifically, as shown in Figure 9, our abstraction engine takes as input a detailed reaction-based model and a set of abstraction properties. The latter help determine which of and how individual abstraction methods should be applied to the input model. These properties can also specify parameters for the conditions used by individual methods, enabling users to control the level of abstraction. The abstraction engine then passes this information through three internal stages: (*i*) pre-processing; (*ii*) main abstraction loop; and (*iii*) post-processing. Pre-processing is used to modify the structure of the input model so that the appropriate abstraction methods in the main loop can be applied more effectively. For example, if a model initially contains irrelevant reactions with respect to a particular species or dynamical property that the user is interested in analyzing—these reactions are removed at the pre-processing step to help speed up the abstraction process. The main loop contains abstraction methods that are applied repeatedly until the structure of the model no longer changes. In the case of gene regulatory networks, abstraction methods such as operator site reduction are typically placed in the main loop. Post-processing is used to transform the model into a form suitable for subsequent application of follow-up analysis methods—e.g., stochastic simulation, Markov chain analysis, etc.

### Abstracted Fimbriation Switch Circuit Model

As discussed earlier, transforming a detailed biological system model into an abstracted one can substantially increase the efficiency of its computational analysis as well as potentially improve our understanding of its overall structure and function. In this work, we have used the reb2sac automated abstraction tool to simplify the detailed model by systematically going through the *fim* switch network and applying various qualifying simplifications and/or approximations as appropriate. The resulting abstracted model is indeed significantly simpler computationally and more understandable logically than the detailed one. For example, the production-degradation reaction scheme of 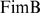 and 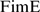 (Figure 5A) is reduced by first quantitatively identifying the transcriptional regulator binding/unbinding events at the *fimB* and *fimE* promoter sites as “rapid” and the corresponding number of the operator sites (one) as “low”—and by then applying the rapid-equilibrium and quasi-steady-state approximations to these processes. The tool then continues to examine the dynamics of other species and finds that the concentrations of 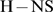 and RNA polymerase (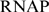) do not change over time in our model. Thus, by applying modifier constant propagation, 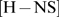 and 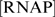 are replaced with constants whose values are set to the corresponding initial concentrations and species 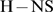 and 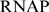 are removed from the model. This process continues until no further reductions are possible.

Taken together with the constraints imparted by the rates involved and the set target of *fim* switching probability, these abstractions reduce the detailed subnetwork of 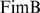 and 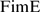 shown in Figure 5A to the one shown in Figure 5B. Similar computational and logical complexity reduction is also achieved for the *fim* element configuration subnetwork. For instance, the reaction process corresponding to the *fim* switch inversion through state 6 (see Figure 4) is given in Figure 6A. The corresponding abstracted reaction scheme is shown in Figure 6B. Overall, after applying all of the available and appropriate abstraction techniques listed in Table 1, the detailed model with 52 reactions and 31 species (e.g., two recombinases, global regulatory proteins, and various intermediate complexes given in Figures 3 and 4) is transformed by reb2sac into an abstracted model with 10 reactions and 3 species (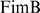, 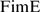, and *switch* given in Figures 5B and 6—the latter showing only reactions involved in ON-to-OFF switching events through circuit state 6).

## Supporting Information

Text S1 Additional modeling information.
(0.21 MB PDF)
