# Supplementary material for: Temperature Control of Fimbriation Circuit Switch in Uropathogenic Escherichia coli: Quantitative Analysis via Automated Model Abstraction
Source: PLoS Comput Biol. 2010 Mar 26;6(3):e1000723. doi: 10.1371/journal.pcbi.1000723 (PMC2845655; doi:10.1371/journal.pcbi.1000723)
Supplement: Text S1 — Additional modeling information. (0.21 MB PDF) [file pcbi.1000723.s001.pdf]

Additional Modeling Information

Temperature Control of Fimbriation Circuit Switch in Uropathogenic *Escherichia coli*: Quantitative Analysis via Automated Model Abstraction

Hiroyuki Kuwahara, Chris J. Myers, and Michael S. Samoilov

General Modeling Assumptions

Aside from the basic assumptions of discrete-stochastic chemical kinetics [1], we have introduced several additional constraints based on the biological properties of the system. Specifically, in our model *E. coli* is assumed to closely resemble a 2μm-long cylinder with 1μm cross-sectional diameter in minimal medium conditions. Thus, the concentration of one molecule inside an *E. coli* cell is set at 1nM throughout. Additionally, the amount of leucine affecting Lrp binding affinities to *fimS* is considered to be in the saturation limit, which enables us to model the dynamics of only activated Lrp form.

Modeling of Temperature-dependent FimB and FimE Regulation

The basic reaction-level subnetwork of FimB and FimE regulation is given in Figure 3. The main mode of temperature control in this process is enabled via a small protein H-NS, which represses the expression of both *fimB* and *fimE* by occupying DNA regions containing *fimB* and *fimE* promoters and preventing RNA polymerase (RNAP) from binding [2]. It has been reported that the *hns* gene is auto-regulated with [H-NS] generally remaining constant, except during certain specific conditions, such as cold shock [3]. Importantly, however, H-NS DNA binding affinity *is* controlled by the ambient temperature and, consequently, so is the production of FimB and FimE [3, 4]. Furthermore, this temperature-dependent transcriptional regulation of *fimB* and *fimE* by H-NS is effected differentially. That is, it has been observed that the expression of *fimB* increases nearly two-fold (119 vs. 195 Miller units) as temperature increases from 30 °C to 37 °C, while the expression of *fimE* decreases about four-fold (226 vs. 61 Miller units) under the same conditions [2].

H-NS rate constants for binding and unbinding to $\mathrm{P_B}$ (i.e., k_2 and k_{-2}) were inferred on the bases of previously reported experimental results as follows. First, we noted that in the case of chromosomal DNA binding: $[\mathrm{P}*] = 1$. That is, for a given level of bound promoter [P∗-H-NS] (which, in turn, sets the level of downstream switching), $K_D \sim$ [H-NS]. So, we next used the empirical temperature-dependent, but *non-specific* K_D^{ns} values for binding of H-NS to DNA at 28 °C, 37 °C, and 42 °C [4] to estimate the amount of $[\text{H-NS}]_{ns}$ that would be required in this case to generate switching probabilities consistent with those measured experimentally [5]. Finally, we deduced the correct (specific) K_D values by multiplying K_D^{ns} by the ratio $[\text{H-NS}]/[\text{H-NS}]_{ns}$, where the true physiological [H-NS] levels were identified as: 20,000 molecules at 37 °C (as reported in [6] for *E. coli* in exponentially growing phase); 30,000 at 28 °C (based on the observations in [7] that levels of [H-NS] held steady between 23 °C and 30 °C at about 1.5 of those observed at 37 °C); and 18,000 at 45 °C (by interpolating results in [8] on the relative protein expression during heat-shock induction to 50 °C). The H-NS rate constants at $\mathrm{P_E}$ (i.e., k_4 and k_{-4}) are inferred analogously, though—unlike the *fimB* case—the negative modulation by H-NS is increased at higher temperature. These K_D values of H-NS binding to $\mathrm{P_B}$ and $\mathrm{P_E}$ are shown in Table S1. Note that the so obtained values of disassociation constants K_D for specific H-NS binding at P∗ are indeed significantly lower than those reported for non-specific DNA interactions [2] as might otherwise be expected from general considerations. The binding rate constants are derived from these K_D values by assuming a rapid unbinding rate and by setting the unbinding rate constant to $10\mathrm{s}^{-1}$. This rapid unbinding rate constant is set by the fastest timescale in the system in order to capture the fast adaptation of recombinases to

temperature perturbation, which may be crucial to rapidly adjusting the fimbriation levels in response to ambient change [5, 9, 10].

In order to estimate the rate constants for the H-NS bindings to promoter regions of the two recombinases at other temperature points, we have performed an exponential curve fitting based on K_D values at the three temperature points shown in Table S1. The results are shown in Figure S1. These K_D values are utilized in the same way as the first three temperature points to infer the values of k_2 and k_4 at the other 7 temperature points. At 28 °C, *fimE* is estimated to produce 200 proteins in one cell generation, while at 37 °C, it produces 61 proteins. These numbers are chosen to be comparable with the ratio of the *fimE* expression data at 30 °C and 37 °C from [2]. To reduce the FimE production even further at 42 °C, *fimE* is assumed to produce 25 proteins in one cell generation. A complete summary of reaction rate constants involved in the FimE-FimB regulation network is provided in Tables 4 and 5.

In order to further evaluate the robustness and fidelity of described estimates for H-NS binding at the two promoter sites, P_B and P_E, we have used the abstracted model to perform a sensitivity analysis of the ON-to-OFF switching frequency (the main objective variable in the problem) with respect to variations of these parameters and across a range of temperature points (Figure S2). The results show that combinations of up to ±20% perturbations in both $\mathrm{P_E}$-H-NS binding and $\mathrm{P_B}$-H-NS binding constants have limited effects on the predicted levels of total ON-to-OFF switching probability. As our results are also consistent with those observed empirically (Table 2), this supports a conclusion that our estimates of binding rate constants are indeed robust against small perturbations as well as faithful to the underlying process dynamics.

The initial concentration of RNAP is taken to be 30nM, which has been previously established as the physiologically available amount of these holoenzymes in *E. coli* grown in minimal medium, and is the same as the level successfully used in previous work analyzing phage λ developmental decision pathway model [11, 12] The initial concentration of H-NS as well as the RNAP binding and unbinding rate constants for both promoter sites (i.e., k_1, k_{-1}, k_3, and k_{-3}) are derived by assuming 50 percent occupancy of H-NS and 25 percent occupancy of RNAP at $\mathrm{P_B}$ at 37 °C. This configuration is found to be effective for modeling the thermoregulation of *fimB* and *fimE* expression by H-NS.

As reported in [2], the ratio of FimB levels in hns^+ versus hns^- mutants at 37 °C is approximately 2. The value of k_5 was derived by matching this ratio, given that FimB is produced around 200 times in one cell generation at 37 °C. The production rate constant of FimE (i.e., k_6) is chosen to be the same as that of FimB.

The value of the degradation rate constant of FimB (i.e., k_{d1}) is chosen so that its production and degradation reactions equilibrate when the concentration of FimB is 100nM at 37 °C. This number is chosen as the best fit from the range of 1 − 100nM thought to be a reasonable value for [FimB] and [FimE] [13]. The degradation rate constant of FimE (i.e., k_{d2}) is then taken to be the same as that of FimB.

Finally, the average initial concentrations of the two recombinases in the ON state (i.e. before we begin monitoring the *fimS* switch shutdown rate) are determined by first starting in a state without any recombinase activity and running an ODE simulation of the [FimB] and [FimE] regulation model for two cell generations. The concentrations of the two recombinases are then retrieved at the end of

Table S1. Temperature-dependent K_D for H-NS binding to $\mathrm{P_B}$ and $\mathrm{P_E}$ derived from reported data.

Temperature (°C)	$\mathbf{K_D}$ at $\mathbf{P_B}$ (μM)	$\mathbf{K_D}$ at $\mathbf{P_E}$ (μM)
28	8.82	15.0
37	10.0	1.81
42	16.63	0.679

the simulation run for each temperature setting to get concentrations around their steady states. The concentrations of FimB and FimE obtained from this scheme are set as the initial concentrations of our *in silico* ON-to-OFF switching experiments at the corresponding temperature setting.

Modeling of the *fimS* Configuration Dynamics

In order to analyze the fimbriation shutdown model, we examine the ON-to-OFF switching dynamics, Figure 4, through 6 possible transition states (out of the 18 available *fimS* configurations), Table 6. Their equilibrium thermodynamics characteristics are based on those given in Wolf & Arkin [13]. In our *fim* switch configuration model, the *fimS* binding and unbinding rate constants are estimated from the standard free energy relationship, $\Delta G = -RT \ln(k_f/k_r)$ using a rapid unbinding rate constant of 1.0s^{-1}. This unbinding rate constant is chosen so that it is an order of magnitude smaller than that in the FimB and FimE regulation for relatively fast adaptation of the two recombinases to temperature perturbation. Since only states 3-8—where IHF and either recombinase species are bound to the switch DNA region—are configured to invert the *fim* switch from ON to OFF, the values of k_p are set to 0 for states 1-2, and 9-18, while the values of k_p for states 3-8 are derived using our qualitative knowledge on the switching regulation, and chosen so that results from our detailed model fit those observed empirically. For example, since the switching rates are faster when Lrp occupies Lrp-I and/or Lrp-II, but not Lrp-III, the values of k_{p5} is chosen to be much greater than those of k_{p3} and k_{p6}. Also, in our model, the initial concentration of IHF is set to 10nM to match the ON-to-OFF frequency from the experimental observation at 37 °C [5].

The initial concentration of Lrp is modeled as an increasing function of temperature to indirectly capture the upregulation of *lrp* expression at higher temperatures owing to the reduction in H-NS-based thermorepression [4, 13, 14]. In our model, the Lrp DNA-binding configurations are simplified to be in one of three states: (1) no Lrp is bound; (2) 2 molecules of Lrp are bound; and (3) 3 molecules of Lrp are bound. The concentration of Lrp is quantified for each temperature setting based on the tuning mechanism of Lrp as illustrated in Figure S3(a). The concentration of Lrp at 37 °C is chosen to be 5nM as this value is determined to be the physiologic concentration of free Lrp in the cell at 37 °C in [13]. The concentration of Lrp at the other two temperature settings is set so that it qualitatively agrees with observations of the temperature tuning mechanism in [13]. At 28 °C, $[\text{Lrp}]_0$ is set to 2nM, which also serves as the lower bound on [Lrp] at lower temperatures, so that Lrp molecules are unlikely to occupy Lrp-I and Lrp-II, and moreover to prevent Lrp molecules from binding to Lrp-III, while at 42 °C $[\text{Lrp}]_0$ is set to 20nM, so that Lrp molecules are likely to occupy all three Lrp binding sites. Using the concentration of Lrp at these three temperature points, the concentration of Lrp at temperature points higher than 28 °C is obtained via exponential curve fitting as shown in Figure S3(b). A summary of the resulting [Lrp] values at various temperature points is given in Table 7.

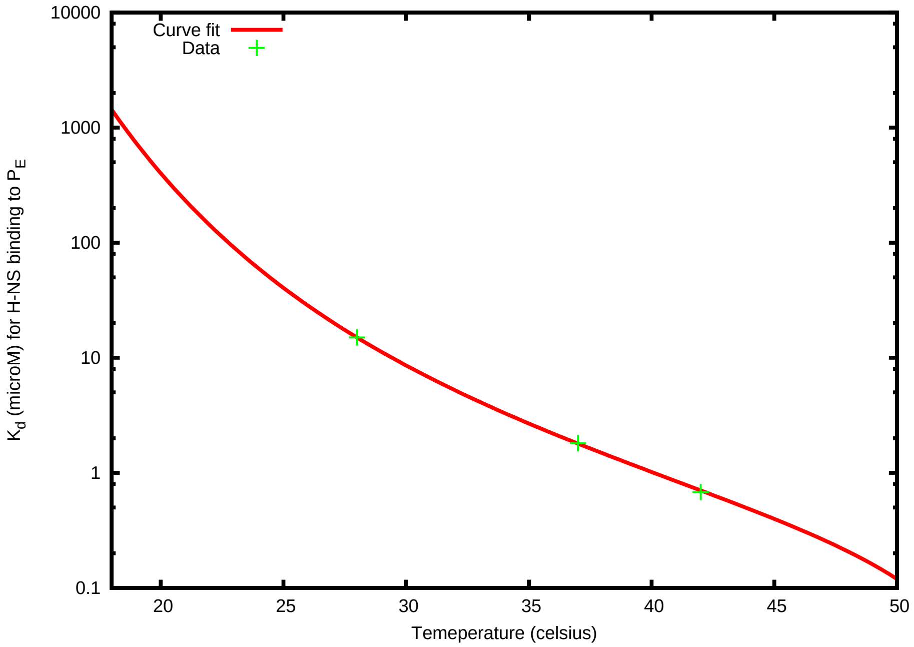

(a)

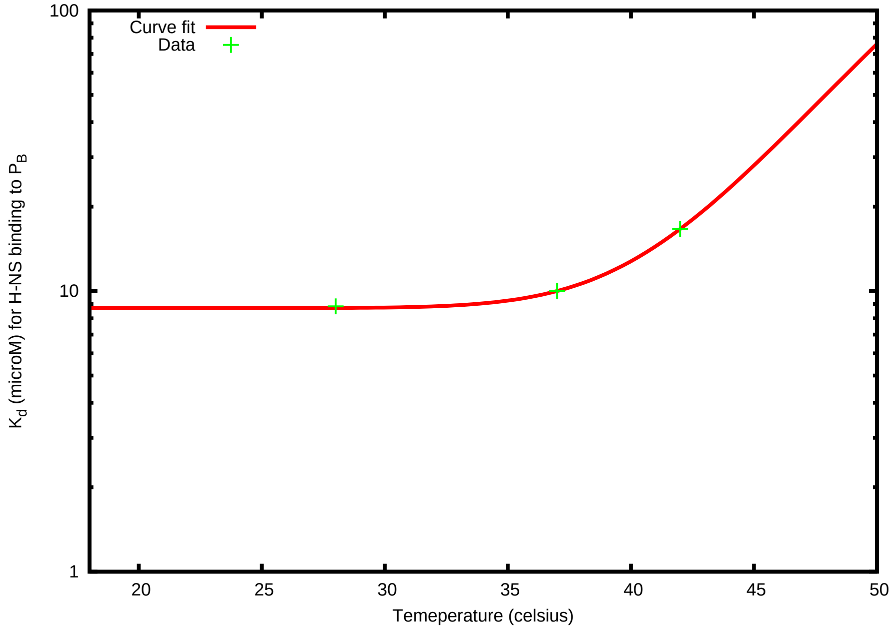

(b)

Figure S1. Estimation of K_D values for H-NS binding to the promoters of the two recombinases at various temperature points using exponential curve-fitting (with the three temperature points at 28 °C, 37 °C, and 42 °C derived from experimental observations, Table S1, taken as input). (a) K_D values of the H-NS binding to *fimE* promoter versus temperature, and (b) K_D values to *fimB* promoter versus temperature.

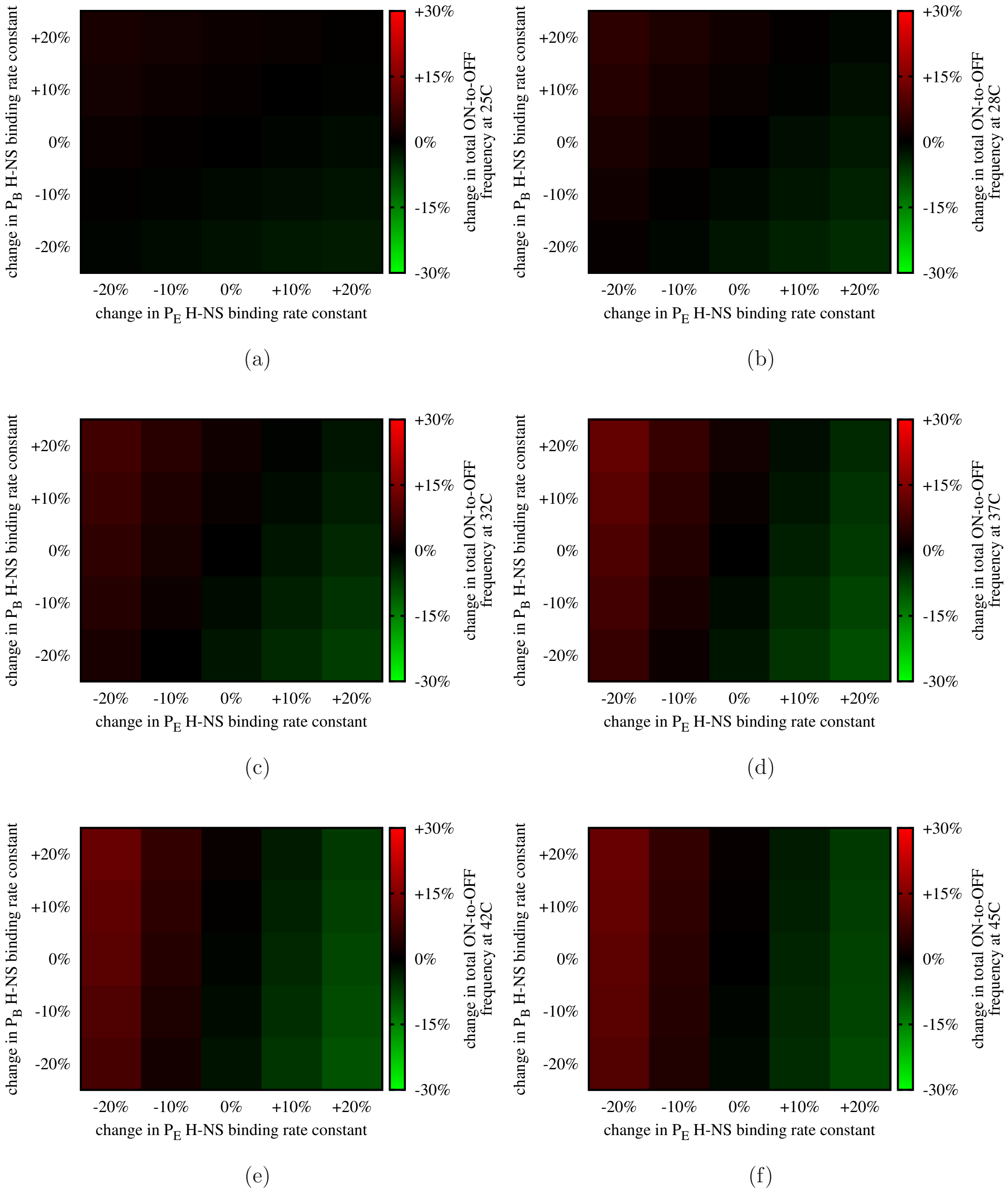

Figure S2. Sensitivity analysis of the ON-to-OFF switching probability against H-NS binding rate constant to the two promoters for various temperature points.

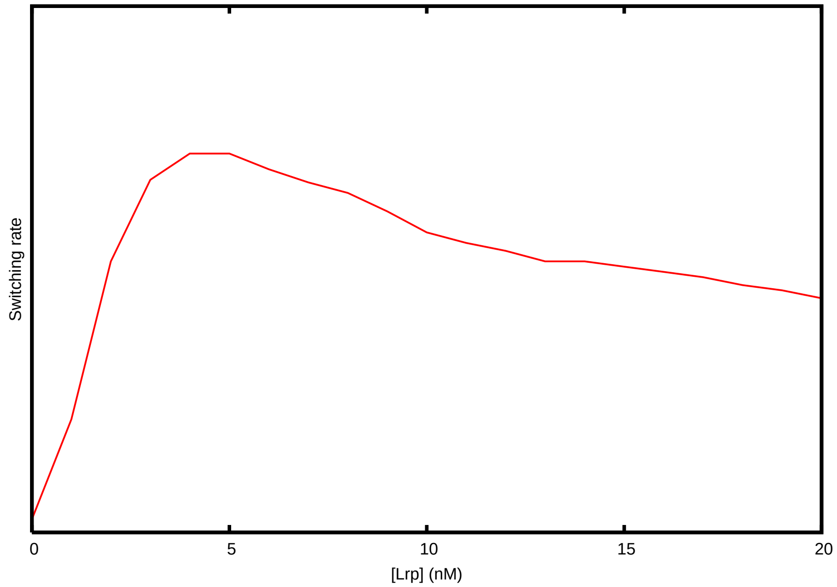

(a)

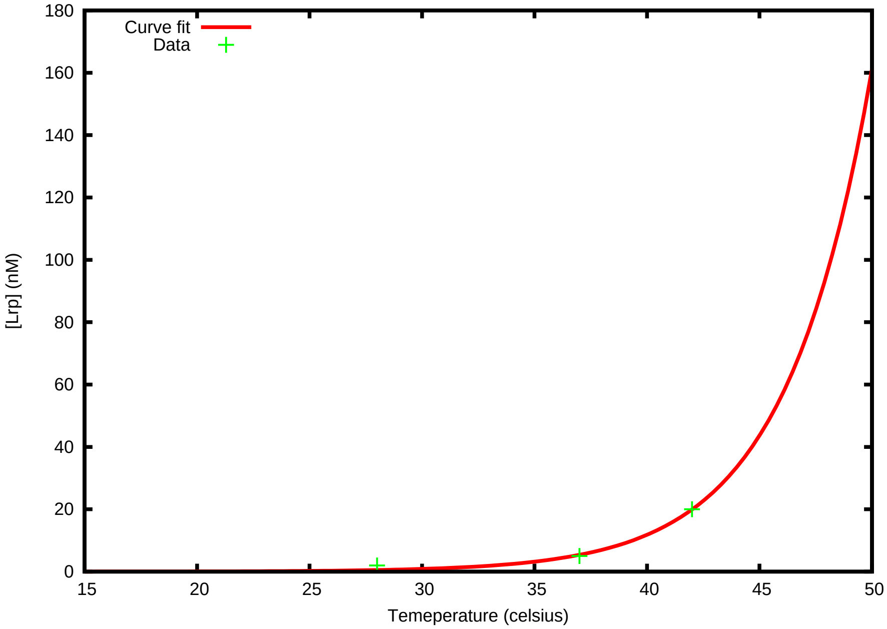

(b)

Figure S3. Temperature tuning mechanism of Lrp. (a) At very low concentration Lrp is unlikely to occupy the Lrp binding sites and the *fim* switching rate is low [13]. When the concentration is around 5nM, Lrp tends to occupy Lrp-1 and/or Lrp-2, but not Lrp-3—the configuration which activates *fimS* switching. As the concentration of Lrp increases even further, Lrp is likely to occupy Lrp-3 as well as Lrp-1 and Lrp-2, which inhibits switching [15]. (b) Estimation of $[\mathrm{Lrp}]_0$ at temperature points higher than 28 °C using exponential curve-fitting (with the three temperature data points at 28 °C, 37 °C, and 42 °C as input).

References

1. Gillespie DT (2005) Stochastic chemical kinetics. In: Yip S, editor, Handbook of Materials Modeling, Springer. pp. 1735-1752.

2. Olsen PB, Schembri MA, Gally DL, Klemm P (1998) Differential temperature modulation by H-NS of the *fimB* and *fimE* recombinase genes which control the orientation of the type 1 fimbrial phase switch. FEMS Microbiol Lett 162: 17-23.

3. Atlung T, Ingmer H (1997) H-NS: a modulator of environmentally regulated gene expression. Mol Microbiol 24: 7-17.

4. Ono S, Goldberg MD, Olsson T, Esposito D, Hinton JCD, et al. (2005) H-NS is a part of a thermally controlled mechanism for bacterial gene regulation. Biochem J 391: 203-213.

5. Gally DL, Bogan JA, Eisenstein BI, Blomfield IC (1993) Environmental regulation of the *fim* switch controlling type 1 fimbrial phase variation in *Escherichia coli* K-12: effects of temperature and media. J Bacteriol 175: 6186-6193.

6. Azam TA, Iwata A, Nishimura A, Ueda S, Ishihama A (1999) Growth phase-dependent variation in protein composition of the *Escherichia coli* nucleoid. J Bacteriol 181: 6361-6370.

7. White-Ziegler C, Angus Hill M, Braaten B, van der Woude M, Low D (1998) Thermoregulation of *Escherichia coli pap* transcription: H-NS is a temperature-dependent DNA methylation blocking factor. Mol Microbiol 28: 1121-1137.

8. Richmond C, Glasner J, Mau R, Jin H, Blattner F (1999) Genome-wide expression profiling in *Escherichia coli* K-12. Nucleic Acids Res 27: 3821-3835.

9. Dorman CJ, Bhriain NN (1992) Thermal regulation of fimA, the *Escherichia coli* gene coding for the type 1 fimbrial subunit protein. FEMS Microbiol Lett 99: 125-130.

10. Chu D, Blomfield IC (2007) Orientational control is an efficient control mechanism for phase switching in the *E. coli* fim system. J Theor Biol 244: 541–551.

11. Arkin A, Ross J, McAdams H (1998) Stochastic kinetic analysis of developmental pathway bifurcation in phage λ-infected *Escherichia coli* cells. Genetics 149: 1633–1648.

12. Kuwahara H, Myers C, Samoilov M, Barker N, Arkin A (2006) Automated abstraction methodology for genetic regulatory networks. Trans on Comput Syst Biol VI: 150-175.

13. Wolf DM, Arkin AP (2002) Fifteen minutes of *fim*: Control of type 1 pili expression in *E. coli*. OMICS 6: 91–114.

14. Oshima T, Ito K, Kabayama H, Nakamura Y (1995) Regulation of *lrp* gene expression by H-NS and Lrp proteins in *Escherichia coli*: dominant negative mutations in *lrp*. Mol Gen Genet 247: 521-528.

15. Kuwahara H, Myers C, Samoilov M (2006) Abstracted stochastic analysis of type 1 pili expression in *E. coli*. In: The 2006 International Conference on Bioinformatics and Computational Biology (BIOCOMP'06). CSREA Press, pp. 125-131.
